# Materials for Photovoltaics: State of Art and Recent Developments

**DOI:** 10.3390/ijms20040976

**Published:** 2019-02-23

**Authors:** José Antonio Luceño-Sánchez, Ana María Díez-Pascual, Rafael Peña Capilla

**Affiliations:** 1 Department of Analytical Chemistry, Physical Chemistry and Chemical Engineering, Faculty of Sciences, Alcalá University, 28871 Madrid, Spain; jose.luceno@uah.es; 2 Department of Signal Theory and Communication, Polytechnic High School, Alcalá University, 28871 Madrid, Spain; rafa.pena@uah.es

**Keywords:** photovoltaics, generations, polymers, carbon nanotubes, graphene, efficiency

## Abstract

In recent years, photovoltaic cell technology has grown extraordinarily as a sustainable source of energy, as a consequence of the increasing concern over the impact of fossil fuel-based energy on global warming and climate change. The different photovoltaic cells developed up to date can be classified into four main categories called generations (GEN), and the current market is mainly covered by the first two GEN. The 1GEN (mono or polycrystalline silicon cells and gallium arsenide) comprises well-known medium/low cost technologies that lead to moderate yields. The 2GEN (thin-film technologies) includes devices that have lower efficiency albeit are cheaper to manufacture. The 3GEN presents the use of novel materials, as well as a great variability of designs, and comprises expensive but very efficient cells. The 4GEN, also known as “inorganics-in-organics”, combines the low cost/flexibility of polymer thin films with the stability of novel inorganic nanostructures (i.e., metal nanoparticles and metal oxides) with organic-based nanomaterials (i.e., carbon nanotubes, graphene and its derivatives), and are currently under investigation. The main goal of this review is to show the current state of art on photovoltaic cell technology in terms of the materials used for the manufacture, efficiency and production costs. A comprehensive comparative analysis of the four generations is performed, including the device architectures, their advantages and limitations. Special emphasis is placed on the 4GEN, where the diverse roles of the organic and nano-components are discussed. Finally, conclusions and future perspectives are summarized.

## 1. Introduction

Electricity is a core resource for the development of human civilizations, and it is possible to link the living standard and the electricity consumption of a society. Electricity can be obtained from diverse resources and with different production methods, ranging from the combustion of raw materials (such as coal, natural gas, biomass, etc.) to complex nuclear reactors systems.

**Figure 1:**
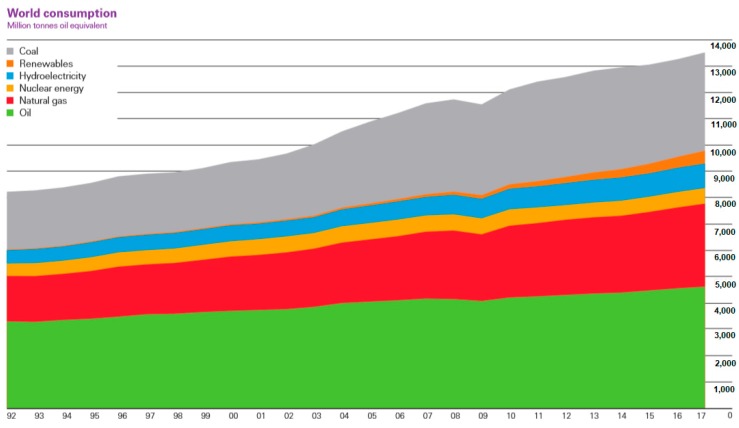
World energy consumption. Taken from [1]

Over the last 50 years, electricity production has continually increased, with a strong presence of fossil fuels [1] (see Figure 1). However, with concern about climate change nowadays, the production must be reoriented towards renewable resources [2,3], such as solar energy, to the detriment of other fossil energies such as coal. The use of this primary energy source entails not only serious polluting emissions, but also a very high consumption of water, at a time when the scarcity of this element has become, for many countries, a key issue of concern.

Solar energy is the energy obtained from solar radiation, and it is regarded as renewable since the Sun expected life is still between 5000 and 10,000 billion years; furthermore, this kind of energy is available in most of the Earth places. Photovoltaic energy (PV) is the electric energy produced directly from the sun radiation by applying the photovoltaic effect [4], which was discovered in 1839 by the French physicist Alexandre-Edmond Becquerel. This effect is found in semiconductor materials, characterized by their intermediate in electrical conductivity between a conductor and an insulator. When the incident radiation in the form of photons reaches the material, these are captured by electrons, resulting in higher energy content, and if a threshold value called “band gap” is exceeded, they can break their nucleus links and circulate through the material. This electron flow generates a difference of potential between the terminals, and upon application of an electric field on the semiconductor, the electrons move in the direction of the field, generating an electrical current [4].

**Figure 2:**
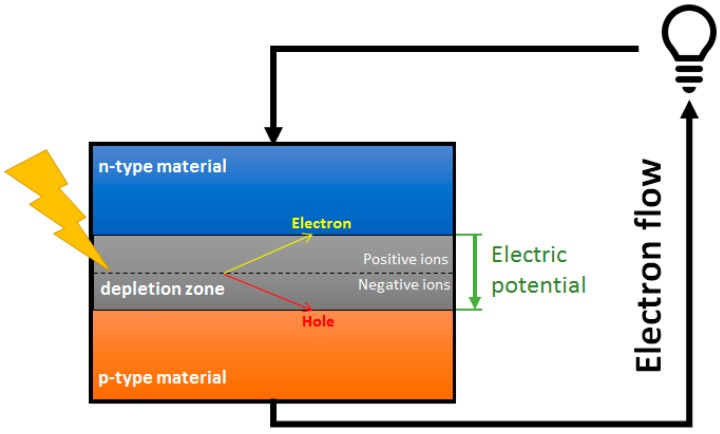
Schematic representation of a photovoltaic cell, showing the n-type and p-type layers.

Photovoltaic cells (PVCs) are devices used to convert solar radiation into electrical energy through the photovoltaic effect. PVCs present an architecture based on the union of two semiconductor regions with different electron concentration (Figure 2); these materials can be type n (semiconductors with excess of electrons) or type p (semiconductors with an excess of positive charges, called holes), though in both cases the material is electronically neutral. 

When both p and n regions are in contact, holes flow from the p region and electrons from the n region through the p-n junction (diffusion current). In addition, the fixed ions near the junction generate an electric field in the opposite direction to the diffusion, which leads to a drift current. At equilibrium, the diffusion current is balanced with the drift current, so that the net current is zero. In this condition, a potential barrier is established at the p-n junction.

As the light strikes the cell, the energy contribution of the photons can be absorbed by the electrons, which can break their bonds, producing hole-electron pairs. These charge carriers are pushed by the electric field and conducted through the p-n junction. If an external load is connected, an electric current and a potential difference between the cell terminals will be established.

**Figure 3:**
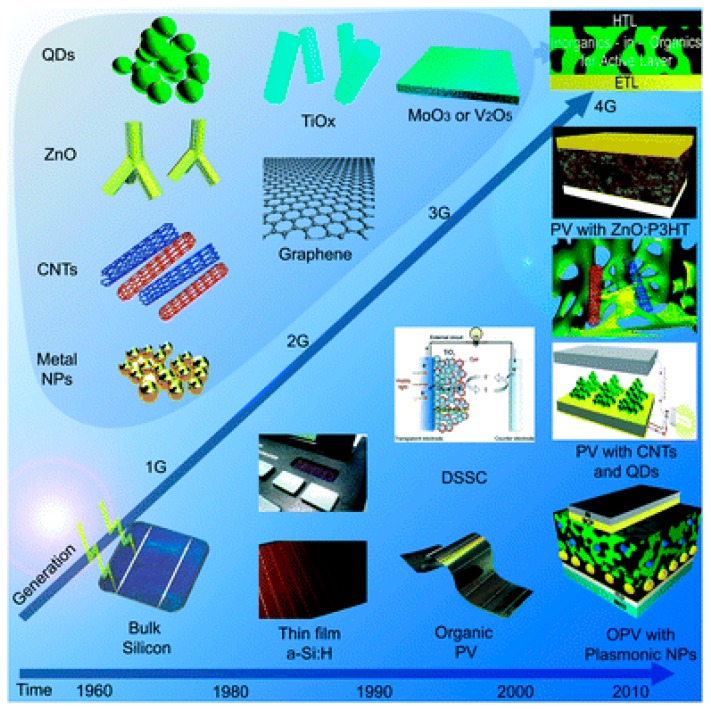
Timeline of the four GEN of photovoltaic cells with the associated materials that comprise each generation. Taken from [5].

The different PVCs that have been developed up to date can be classified into 4 main categories called generations [5] (Figure 3):

- First-generation (1GEN): It is based on crystalline silicon technologies, both monocrystalline and polycrystalline, and on gallium arsenide (GaAs);
- Second-generation (2GEN): It includes amorphous silicon (a-Si) and microcrystalline silicon (μc-Si) thin films solar cells, cadmium telluride/cadmium sulfide (CdTe/CdS) and copper indium gallium selenide (CIGS) solar cells;
- Third-generation (3GEN): It involves technologies based on newer compounds including nanocrystalline films, active quantum dots, tandem or stacked multilayers of inorganics based on III–V materials, such as GaAs/GaInP, organic (polymer)-based solar cells, dyed-sensitized solar cells, etc.; and
- Fourth-generation (4GEN): Also known as “inorganics-in-organics”, it combines the low cost/flexibility of polymer thin films with the stability of novel inorganic nanostructures such as metal nanoparticles and metal oxides or organic-based nanomaterials like carbon nanotubes, graphene and its derivatives.

**Figure 4:**
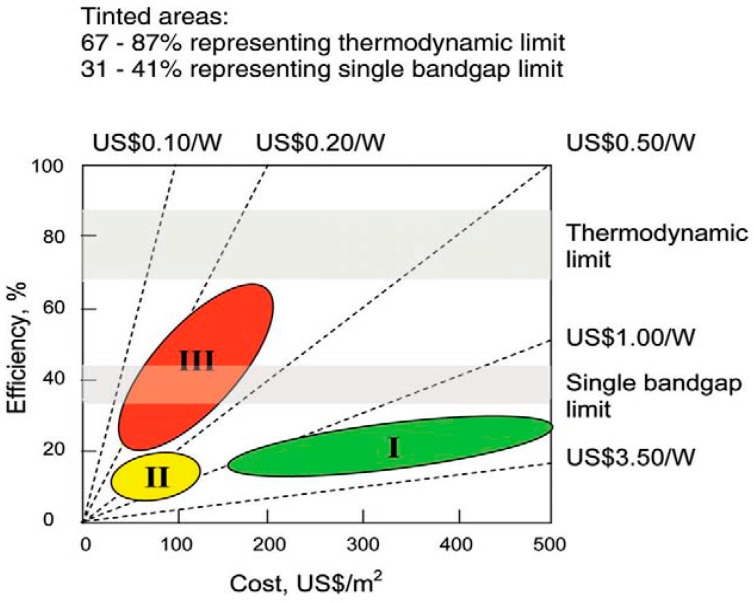
Efficiency and cost projections for first-(I), second-(II), and third-(III) generation PV technologies. Taken from [6].

**Figure 5:**
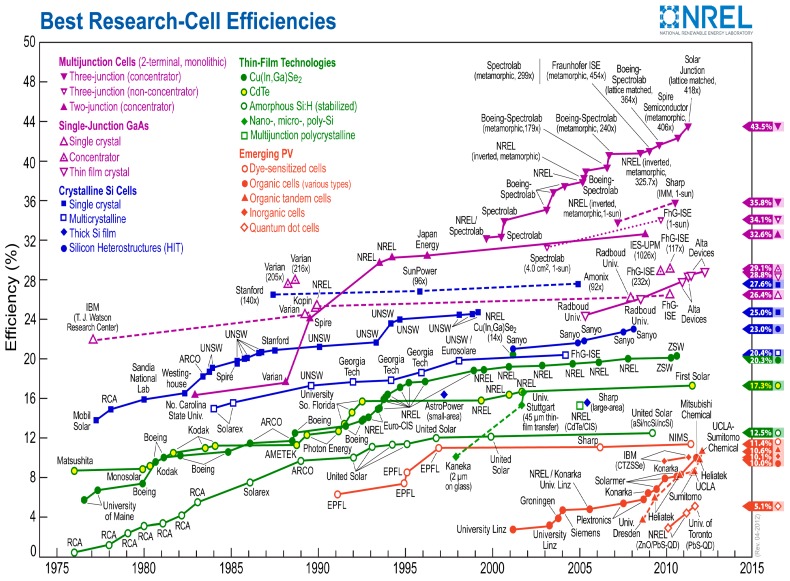
Best research-cell efficiencies. Taken from [10].

Figure 4 shows a diagram of the three first generations of PVCs in terms of their costs and efficiencies [6], and Figure 5 shows the best research efficiencies attained for the different types of solar cells. The aim of each generation is to reduce costs and simultaneously improve efficiency compared to the previous one(s). In this regard, calculations as well as economic and funding feasibility features should be made prior to the design of a PV system [7]. On the other hand, there is lack of agreement in the literature regarding the classification of PVCs, and several authors sort them into different generations like occurs with GaAs and polycrystalline silicon [8] or silicon nanotubes [8,9]. In addition, there is controversy concerning the existence of the 4GEN, since some authors include it in the forefront of the 3GEN [6], while others believe that it is a different one [5,8]. 

The aim of this article is to illustrate the current state of art on photovoltaic cell technology in terms of the materials used for the device fabrication, its efficiency and associated costs. A detailed comparative analysis on the four solar cell generations is performed, focusing on the different architectures, their benefits and drawbacks. Particular attention is placed on the 4GEN, where the functions of the organic and nano-components will be depicted, together with the difficulties related with the fabrication of such devices. A summary and a future outlook will be presented in the last section.

## 2. First-Generation Photovoltaic Solar Cells

The 1GEN comprises photovoltaic technology based on thick crystalline films, namely cells based on Si, which is the most widely used semiconductor material for commercial solar cells (~90% of the current PVC market [8]), and cells based on GaAs, the most commonly applied for solar panels manufacturing. These are the oldest and the most used cells due to their reasonably high efficiencies, albeit are relatively expensive to produce.

The use of Si in the PVC production has some advantages:

- It is the second most abundant material in the earth’s crust [11], which implies that the availability of the raw material may be durable in the future and its acquisition cost could be reduced;
- It is a stable and non-toxic chemical element, characteristics that delay the contamination processes and the loss of durability that may occur when used as a cell material; and
- Si PVCs are easily compatible with Si-based microelectronics industry (i.e., integrated circuits, transistors, etc.) [8], thus allowing the use of well-known and well-developed technologies.

On the other hand, GaAs is especially useful for multi-junction cells, those comprising multiple p–n junctions made of different semiconductor materials, and high-performance PVCs for several reasons [12]:

- It has a band gap of 1.43 eV, which is quite close to the ideal value for single-junction PVCs;
- It presents a very high absorptivity, hence a cell of only a few microns thick is enough to absorb the usable sunlight spectrum corresponding to its band gap, while crystalline Si requires cells of 100 microns or even thicker;
- It allows a versatile cell design, since the incorporation of different dopant substances and the combination with other III-V materials within the cell structure significantly change the optoelectronic properties;
- It is very resistant to radiation degradation, which combined with its high efficiency makes it ideal for space applications; and
- Unlike Si-based cells, those based on GaAs have low temperature coefficients, thus their performance is less affected by temperature.

1GEN PVCs can be further divided into three categories: monocrystalline and polycrystalline Si, as well as GaAs cells.

### 2.1. Monocrystalline Silicon (m-Si)

The m-Si technology has achieved efficiencies of the order of 24.4% [13]. The m-Si cells are manufactured by the Czochralski process [8], which consists in the growth of Si ingots from small monocrystalline silicon seeds [14], and subsequently cut them to obtain m-Si wafers. This process allows the production of crystals with diameters ranging from 10 up to 300 mm, and lengths from 50 cm up to 2 m [14,15]. However, the Czochralski process involves a high production cost owing to several reasons:

- It requires Si of very high purity (known as solar grade silicon) to avoid contamination by the raw material [8], since it would lead to defects in the structure and worsening of the electric properties;
- Energy consumption is high, due to heat losses via conduction and radiation through the seed [8]; and
- Temperature must be controlled to maintain the crystal growth during the long production times [8].

### 2.2. Polycrystalline Silicon (p-Si)

The p-Si cells are manufactured on polysilicon wafers, which consist of small Si crystals randomly oriented. They present several advantages compared to the m-Si ones: they involve less energy in their production and less associated greenhouse effects. However, this type of PVCs can only reach efficiencies of 19.9% [13], smaller than m-Si based cells.

The main reason for this smaller efficiency is the lower material quality due to grain boundaries and defects, and the higher concentration of impurities. Therefore, the influence of recombination in p-Si cells is higher than in m-Si cells, which leads to a slightly lower voltage. Current is also lower due to incomplete carrier collection in these devices. 

p-Si can be obtained at an industrial scale by the Siemens process, initially developed for electronic applications in the 1950s [16]. In general, the process consists in a gasification of the metallurgical grade Si, a distillation of the product, and a final deposition to obtain ultrapure silicon.

### 2.3. Gallium Arsenide (GaAs)

GaAs cells currently reach efficiencies in the range of 18.4–28.8% in the laboratory, depending on whether they have a crystalline structure or they consist in a thin layer [13].

GaAs is obtained by direct combination of Ga and As via a vapour-phase reaction at low pressure and high temperature. The production of GaAs can be summarized into 4 stages: growth of the ingot, wafer processing, epitaxy, and manufacture of the device [17,18]. 

In the first step, a mixture of Ga, As, and small amounts of dopant materials (Si, Te, Cr) are reacted under high temperature conditions to form crystalline ingots of doped GaAs. The processing consists in characterizing the structure of the GaAs by means of X-rays, and in eliminating the ends of the ingot with a diamond saw. Subsequently, the ingot is separated in wafers with a saw, its surface is washed with strong acids (H_2_SO_4_) or H_2_O_2_/H_2_O mixture, cleaned in a lapping machine, rinsed with a soapy solution, dried and finally polished with a polisher. The GaAs wafers are used as a substrate for the growth of very thin sheets of the same or another compound (belonging to groups III–V) possessing the desired electronic or optical properties. This growth is carried out by epitaxy, a process in which the crystallinity of the substrate (GaAs) conditions the crystalline growth of the compound that is incorporated on top [19]. Epitaxy can be performed following different techniques:

- In vapour phase (VPE): a stream of heated vapor from the material interacts with the surface of the wafer;
- In liquid phase (LPE): a hot and saturated metal solution is brought in contact with the wafer; and
- In metalorganic vapour-phase epitaxy (MOCVD or OMVPE): the growth of the crystals is produced by a chemical reaction between the surface and the compound to be deposited. Currently, it is the most used at an industrial level.

Finally, the device manufacture consists in the incorporation of the metallic contacts and the antireflective layer, the isolation of the devices, their encapsulation the cell and other auxiliary processes.

One of the main advantages of GaAs for PVC applications is that it offers a wide range of potential design options. GaAs-based cells can have several layers with a slightly different composition that allow a more accurate control of the generation and collection of electrons and holes than silicon cells, which are limited to changes in the level of doping to achieve the same results. This higher degree of control allows to achieve efficiencies closer to the theoretical limit. For instance, one of the most common GaAs cell structures has a very thin window layer of AlGaAs that enables electrons and holes to be generated close to the electric field at the junction. Further, GaAs is frequently used in multi-junction solar cells, where each p-n junction produces an electric current in response to different wavelengths of light. The use of multiple semiconducting materials allows the absorption of a broader range of wavelengths, thus improving the energy conversion efficiency of the cell [20]. 

## 3. Second-Generation Photovoltaic Solar Cells

The 2GEN focuses on thin-film technologies with the aim of reducing the high costs associated with the 1GEN by using lower amount of material and of poorer quality, deposited on cheap substrates. It is based on materials identified as potentially useful during the development of the 1GEN and was extended to include a-Si, μc-Si, CIGS, and CdTe [5]. 

The 2GEN PVCs present the following general advantages [20,21]:

- Cheaper compared to Si-based solar cells
- Drastic reduction in the amount of materials needed. Sometimes only one-micron thick layer is required.
- High absorption coefficient.
- Can use both vacuum and non-vacuum process.
- Most of technologies allow direct integration into a higher voltage module (i.e., a-Si), which reduces the number of production stages compared to the 1GEN-PVCs.

However, they also present some disadvantages:

- Lower efficiency: the best efficiency reached in the laboratory is 20.3% for CIGS [10].
- Light-induced degradation in first stages of outdoor usage. Higher degradation in outdoor uses: the semiconductor deposited onto glass can generate a flow of ions in the glass. In the case of amorphous silicon, this problem can occur even if the substrate is not glass. Environment contamination starts from fabrication process.
- In some technologies, the availability of manufacturing materials may not be abundant.

### 3.1. Amorphous Silicon (a-Si) and Micro-Crystalline Silicon (μc-Si)

a-Si is widely used in thin-film solar cells owing to several reasons [22]:

- Raw materials are abundant and non-toxic;
- It requires low-temperature processes, allowing the manufacturing of modules with a wider and a cheaper range of substrates;
- It presents a high absorption coefficient, hence cells are thinner (of the order of 1–2 μm thick) and require less amount of material per cell;
- Large area deposition technologies can be applied [23];
- Laboratory efficiencies of a-Si are 10.2% for single-junction cells and 12.7% for multi-junction cells [13].

However, a-Si also presents some limitations:

- The non-crystalline structure of a-Si reduces the life cycle, due to the formation of hole-electron recombination centres; and
- The absence of crystalline structure hinders doping treatments with n-type and p-type compounds. Hydrogen is required to dope the material, leading to hydrogenated amorphous silicon (a-Si:H).

The use of a-Si:H can lead to operational problems since it can be degraded by sunlight: the thickness of the layer would decrease over time, and thicker layers would be required to ensure long-term operation. However, single-junction and multi-junction devices with high efficiency and moderately good stability have been developed [22]. In addition, if the process involves a high concentration of H, μc-Si can be formed, which has fewer defects than a-Si and is more stable in the presence of solar radiation; the lab efficiencies of μc-Si are in the range of 11.9% for single-junction cells and 14.0% for multi-junction cells [13].

**Figure 6:**
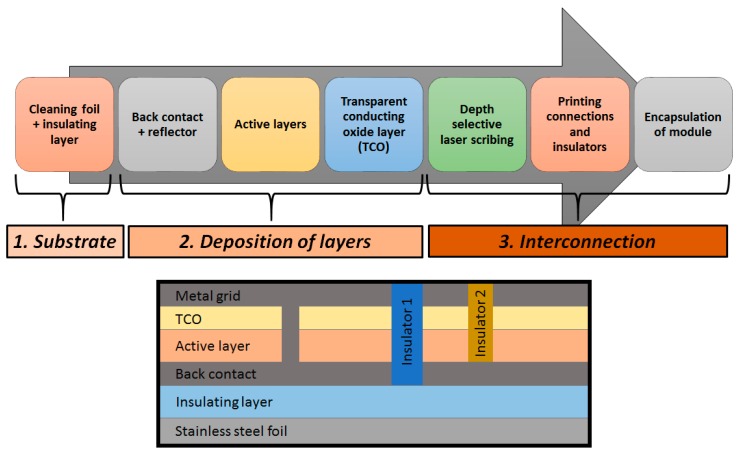
Schematic representation of the manufacturing process of a-Si based photovoltaic cell.

The typical production process of a-Si:H cells is a roll-to-roll process [24], as schematically shown in Figure 6. Firstly, a cylindrical sheet, usually stainless steel, is unrolled to be used as a deposition surface. The sheet is washed, cut to the desired size, and printed with an insulating layer. Then, the a-Si: H is deposited on the reflector. Subsequently, the transparent conductive oxide (TCO) is deposited on the silicon layer. Laser cuts are then made to interconnect the different layers and, finally, the module is encapsulated.

### 3.2. Copper Indium Gallium Selenide (CIGS)

**Figure 7:**
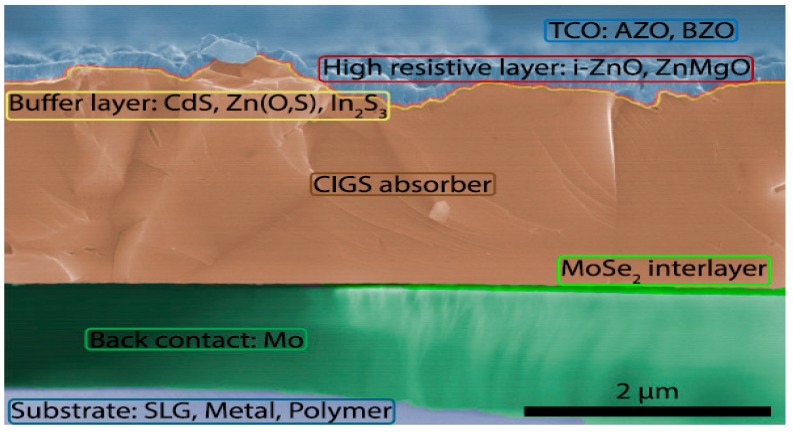
Cross-section of a CIGS cell. Taken from [27].

CIGS is a semiconductor material with general formula of Cu (In_x_Ga_1-x_)Se_2_ that varies its band gap value between 1.0–1.7 eV depending on the proportion of the elements in the compound [25]; it is synthesized by preparing a molten mixture containing the desired amount of each element [26]. CIGS cells are usually manufactured following five steps [27]: (1) A substrate such as Na_2_CO_3_–CaO, a metal, a ceramic or a polymer sheet is placed to support the rest of the cell. (2) The substrate is covered with the back contact, which is usually pulverized molybdenum in the form of MoSe_2_. (3) The CIGS layer (p-type) is grown by a co-evaporation process. (4) A buffer layer (n-type), currently formed by a TCO such as zinc oxide (ZnO) with or without doping is deposited [25]. (5) Finally, an anti-reflective coating is applied to improve the cell efficiency. The cross-section structure of a CIGS cell is depicted in Figure 7.

Although co-evaporation is the most widespread production technique, other techniques such as a one-stage hot wall deposition process are currently being investigated as an alternative to reduce production costs [26]. For instance, the third step can be replaced by a spraying of the CIGS precursor elements followed by selenization and sulphuration, which results in laboratory efficiency of 22.3% [27]. This would aid to reduce the production cost, since the co-evaporation processes require a great deal of energy.

### 3.3. Cadmium Telluride (CdTe)

CdTe is a semiconductor compound with a band gap of 1.45 eV, which makes it a good candidate for converting sunlight into electricity in single-junction cells. CdTe cells achieve lab efficiencies of around 21% [13], and can be obtained mainly by three different routes:

- Direct reaction of Cd and Te at high temperature in a sealed empty quartz tube;
- Exposure of a Cd solution to gaseous H_2_Te under an inert atmosphere; and
- Addition of Cd in an alkaline metal telluride solution.

**Figure 8:**
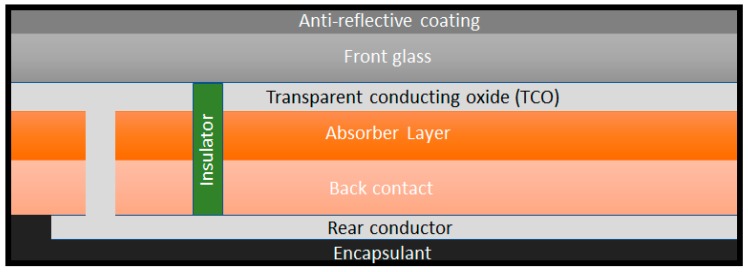
Cross-section of a CdTe-based solar cell.

CdTe cells are manufactured by a multiple deposition process that lasts less than 2.5 h, as schematized in Figure 8: Firstly, a layer of cadmium sulfide (CdS) is vapour deposited onto a transparent conductive oxide film, front contact [−], which is supported on a heat-treated glass. Then, a CdTe layer is deposited on the CdS layer. A laser cut that goes through the three layers is made to introduce the insulator into the module. Afterwards, several cuts are made with the laser, crossing only the CdS and CdTe layers, in order to add the rear contact [+] by sputter deposition and later crossing only the back contact layer. Finally, the cell is encapsulated, the wires are connected and the tempered rear glass is placed.

CdTe PVCs can withstand high temperatures better than c-Si cells and capture radiation better in humid environments. However, the elements that make up the CdTe are scarcer than Si, and CdTe is a potentially toxic material.

## 4. Third-Generation Photovoltaic Solar Cells

The 3GEN arises from the idea of increasing device efficiency and reducing the distance to the Carnot limit, which is ~62% above the Shockley-Queisser limit (33%) [28]. Its aim is to develop devices with high efficiencies using the thin layer deposition techniques employed for the 2GEN and/or new architectures or materials [6]; this may lead to an increment in the area cost, but the cost per watt peak would be reduced. In addition, like Si-based cells, 3GEN-PVCs use non-toxic and very abundant materials, hence are suitable for the large-scale implementation of photovoltaic cells [6]. Further, they may employ new nanostructured or organic materials that could achieve high conversion efficiencies (greater than 60%) using phenomena such as the hot carriers collection [28], the generation of multiple carriers (impact ionization), or new semiconductor architectures that contain multiple energy levels. Considerable attention is paid to charge and energy transfer processes, and routes to optimize charge collection and improve the energy capture within the solar spectrum [29]. 

The most important technologies included in the 3GEN-PVs are:

- Dye-sensitized solar cells (DSSCs);
- Organic and polymeric solar cells;
- Perovskite cells;
- Quantum dot cells; and
- Multi-junction cells.

The main advantages of 3GEN-PVCs are:

- Solution-processable technologies;
- Suitable for large-scale production;
- Mechanical robustness; and
- High efficiencies at high temperatures.

However, their key challenge is reducing the cost/watt of delivered solar electricity. 

### 4.1. Dye-Sensitized Solar Cells

**Figure 9:**
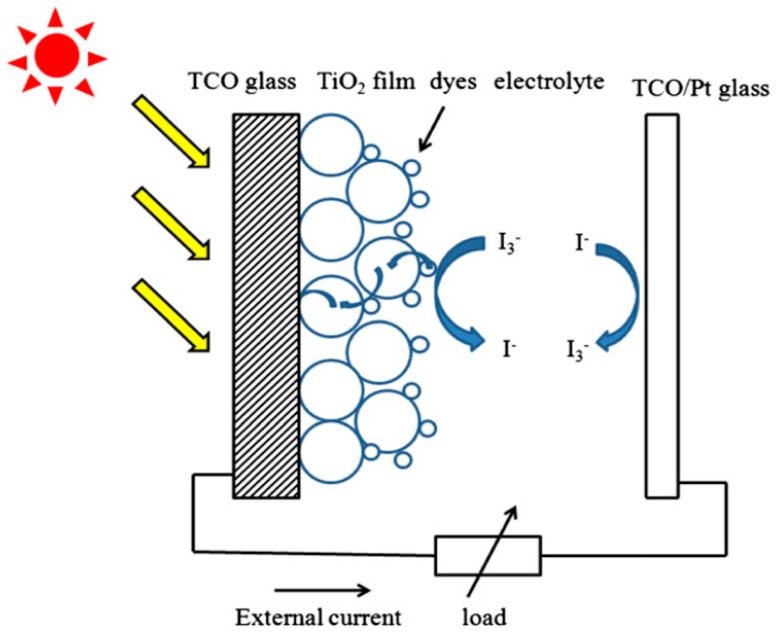
Schematic representation of a DSSC. Taken from [30].

DSSCs are low-cost solar cells in the form of thin films based on a semiconductor formed between a photo-sensitized anode and an electrolyte. They comprise five different layers (Figure 9) [30]:

- A transparent anode manufactured with a glass sheet, treated with a transparent conductive oxide layer (TCO glass);
- A layer of mesoporous oxide (usually TiO_2_) deposited onto the anode to improve electronic conduction;
- A monolayer of charge transfer dye covalently bonded to the surface of the mesoporous oxide layer to enhance the absorption of light;
- An electrolyte containing a redox mediator in an organic solvent, which improves the regeneration of the dye; and
- A cathode made with a crystal coated with a catalyst (usually platinum) to facilitate the collection of electrons.

When the DSSC is exposed to sunlight, the sensitizing dye is excited and an electron moves to the conduction band of the mesoporous oxide film. The dye promotes the diffusion of electrons towards the anode and is then reused in the external charge (anode) before becoming part of the cathode (see the changes experienced by the species I^−^/I_3_^−^ in Figure 9), thus completing the dye cycle.

To improve the electrical conductivity and the capture of light at the back layers, a conductive crystal is used, the most common being tin oxide doped with indium (ITO) and zinc oxide doped with fluorine (FTO). The crystal choice depends on the cell configuration and its materials [31]. The semiconductor electrode is usually a thin-film layer (~5–30 μm) of nanocrystalline titanium dioxide (TiO_2_) deposited onto the conductive glass, and it plays an important role both in the exciton separation and in the electron transfer process. The porosity and the morphology of the TiO_2_ layer are significant factors that condition the amount of dye molecules absorbed on its surface [30], thus allowing to collect more or less incident light.

Since the first DSSC was introduced, many artificial dye molecules have been synthesized, and some of them have been successfully launch into the market such as the so-called N3, N719, and Z907 [30]. The dye molecules must fulfil some requirements, such as matching the solar spectrum, long-term operational stability, and strong attachment to the semiconductor surface. In addition, the redox potential must be high enough to facilitate the regeneration reaction with the redox mediator [32]. This type of cells is ideal in low density applications like rooftop solar collectors, where the mechanical robustness and light weight of the glass-less collector is a major advantage. Conversely, they are not useful for large-scale deployments where higher-cost higher-efficiency cells are more suitable.

DSSCs present a number of advantages; for instance, they work even in low-light conditions, hence are able to work under cloudy skies and indirect sunlight, while conventional designs would experience a “cut-out” at some lower limit of illumination. Another advantage is the cell mechanical robustness that results in higher efficiencies at higher temperatures, whilst traditional silicon cells suffer significant decreases in efficiency as the cells heat up internally. DSSCs typically comprise only a thin conductive plastic as the front layer, which enables a simple and fast dissipation of heat; hence operating at lower internal temperatures.

The foremost disadvantage of the DSSCs is the use of a liquid electrolyte that has temperature stability issues. The electrolyte can freeze at low temperatures, ending power production and frequently resulting in physical damage. Conversely, higher temperatures cause the liquid to expand, making the sealing of panels an important issue. Replacing the liquid electrolyte by a solid is an important field of research. Trials with solidified melted salts are promising, albeit these are not flexible and experience higher degradation during continuous operation.

An additional drawback is that the electrolyte solution contains volatile organic compounds that are toxic to human health and the environment, which combined with the permeability of the solvents through the plastics, has prevented from large-scale outdoor applications and integration into flexible structure.

Advances in DSSCs are mainly based on the following research lines [33]:

- Ruthenium complexes (Ru);
- Organic compounds free from metals;
- Sensitizers made of quantum dots;
- Sensitizers based on perovskite;
- Mordanting dyes and
- Natural dyes.

Ru complexes are classified into Ru polypyridyl carboxylate dyes, Ru phosphonate dyes, and bipyridyl polynuclear Ru dyes [33]. They have enhanced the efficiency from ~7.1% in 1991 to 11.2% in 2005 [34,35], and present a number of advantages including excellent stability, high absorption in the visible spectrum, excellent electron injection, and good charge transport efficiency between the metal and the ligand. However, Ru is toxic and expensive since it is a rare metal, hence current research is aimed at searching for dyes without Ru [33], such as organic dyes without metals and complex metal-porphyrin dyes. Further, Ru is a non-renewable resource and tends to undergo degradation in presence of water, therefore, is of limited use in large-scale applications.

On the other hand, metal-free organic dyes are cheaper and present a wider range of molecular structures. The success of these dyes lies in their tunable physicochemical properties, as well as their good coefficient of extinction in the visible spectrum [36]. They are usually classified into donors, unions or acceptors [33], and achieve energy conversion efficiencies in the range of 5–9%, although the best efficiency attained by union dyes is ~10% [37,38], and that of the best donor reaches 10.3% [39]. In 2011, a method for obtaining metal-free sensitizers with porphyrin dyes was proposed, resulting in a very high efficiency of 12.3% [40]. Numerous porphyrin dyes have been identified as light-gathering components for DSSCs due to their powerful absorption in the visible spectrum and their redox properties for the sensitization of TiO_2_ sheets [33].

DSSCs with quantum dots (QDs) are based on the substitution of the dye by inorganic nanoparticles of quantum dots, which will be described below. This approach is currently being developed since enables to carry out the exciton transition with higher energy and at lower optical transition rates owing to the tunable size, high optical absorption and good magnetic properties of QDs [41]. Most of the research has been performed with cadmium compounds, reaching efficiencies close to 6.76% [42].

DSSCs based on perovskite sensitizers began to be applied in 2009 with efficiencies in the range of 3.1–3.8% [43], although currently the efficiency is close to 20% [33,44], since these sensitizers are excellent light collectors.

Natural dyes are isolated from natural compounds such as fruits, vegetables (i.e., chlorophyll and anthocyanins [45]), bacteria, flowers, etc. Betalain pigments are another class of dyes present in Caryophyllales plants that have been subjected to a detailed photoelectrochemical study since have high molar extinction coefficients in the visible region and pH dependent redox properties [46]. The pigments are present in the different part of the plant including flowers petals, fruits, leaves, stems, and roots. The main advantages of the natural dyes are their absorption in the visible spectrum, easiness of preparation and ecological viability [47,48], together with their low production cost due to the absence of noble metals [33]. The efficiencies obtained using these dyes are low, between 0.03 and 1.50%, although in 2008 a device reached 1.7% [46]. Thus, more research is needed before natural-dyed SSCs can become a cost-effective and environmentally friendly large-scale choice.

Mordant dyes are a type of synthetic colorants applied to textile fibres with an enhanced affinity for the fibres due to the addition of mordants, which are typically compounds such as sodium chloride, chromium alum, or metals such as iron, chromium, copper, etc. [33].

These dyes are much cheaper than Ru complexes, are free from heavy metals and are manufactured at an industrial scale. Millington et al. [49] studied the performance of 49 commercial mordant dyes as sensitizers in DSSCs and compared them to that of N3 ruthenium complex. Although N3 led to the highest output, six mordant dyes produced photocurrents higher than 0.2 mA, and the UV–visible spectra of the dye-complexed photoanodes suggested that some mordant dyes are more strongly bound to the TiO_2_ surface than N3. Further, photocatalytic oxidation of these dyes does not occur in a DSSC environment. Additional research in this direction is required to determine whether the use of purified dye samples would improve their performance for commercial uses. 

### 4.2. Quantum Dot Solar Cells

**Figure 10:**
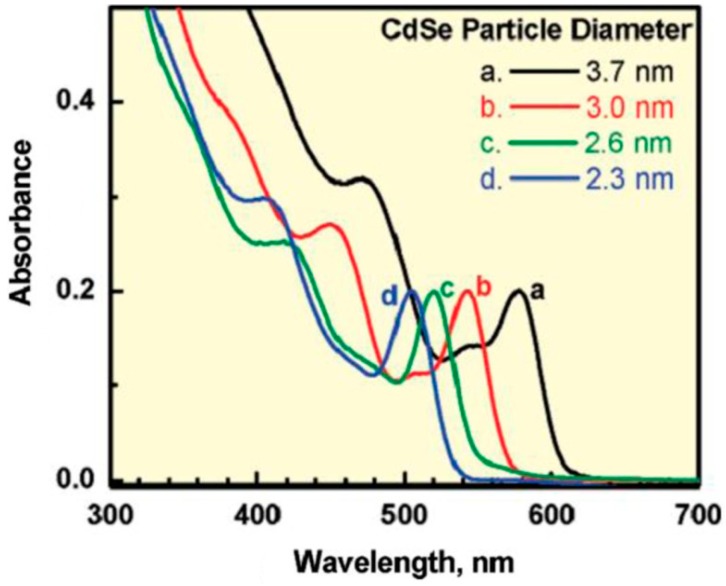
Absorption spectra of CdSe QDs with different diameters. Taken from [51].

Quantum dots (QDs) are nano-scale semiconductor materials, belonging to groups II–VI, III–V, or IV–VI of the periodic table, that have a discrete spectrum of quantized energy, since the movement of the electrons and holes is confined in the three directions of the space. Owing to their nanoscale dimensions, typically between 2–10 nm [50,51], they exhibit properties that are intermediate between those of bulk semiconductors and those of discrete atoms or molecules. In a typical semiconducting material, electrons jump from the valence to the conduction band when energy higher than the band gap is provided. However, owing to the quantum confinement effect, the two bands are so close together that can be considered as continuous bands [50]. Due to the small size of the QDs, the values of these bands are quantized, in a way that a modification of the QDs’ size implies a change in the band gap value and in the absorption spectrum (Figure 10).

Typically, QDs have a structure consisting of a core on which layers of different compounds are deposited with the aim to improve efficiency by reducing interaction forces between the exciton and the surface of the nanoparticle [50]. It is also possible to embed QDs in a matrix of other material.

The conversion efficiency of this type of cells has increased over recent years, being currently above 11% [52]. This value is difficult to be increased due to the diffusion of charge carriers, thus new cell structures are required, or the combination of QDs technology with other types of cells [53,54,55,56,57,58,59]. It is also feasible to increase efficiency through doping with other materials [60]; for instance, Si doping can increase efficiency from 11.3% to 17.0%.

The amount of QDs is another key factor in the transport and recombination of charges, that strongly affects the cell efficiency; thus, variations up to 40% have been reported depending on the QDs concentration [60,61].

As discussed earlier, current QDs research is combined with other technologies. such as DSSCs [60]. In this regard, the addition of QDs has improved the cell efficiency from approximately 1% to 11.6% [10,62,63,64]. Nonetheless, the theoretical efficiency limit of QDs technology is estimated to be 63% [65].

Overall, the main potential benefits of QDs-based PVCs are [66,67]:

- Favorable power to weight ratio;
- High efficiency;
- Mass and area savings as well as flexibility leads to miniaturization;
- Lower power consumption;
- Versatility;
- Increased electrical performance at low production costs; and
- Can be used in complete buildings including windows and not just rooftops.

It is important to note that this technology is still at the laboratory scale, hence the indicated advantages need to be tested in an industrial context.

Conversely, the drawbacks of these cells are derived from the technological immaturity and the lack of experience in the development and manufacturing processes. Further, some QDs are highly toxic in nature (i.e., CdSe) and require very stable polymer shell [68]. Additionally, shells can alter optical properties, and degradation increases under aqueous and UV conditions. The particle size is also difficult to control.

### 4.3. Organic and Polymer Solar Cells

Organic photovoltaic cells (OPVCs) are those that use conductive organic polymers or small organic molecules for light absorption and charge transport to produce electricity from sunlight. 

**Figure 11:**
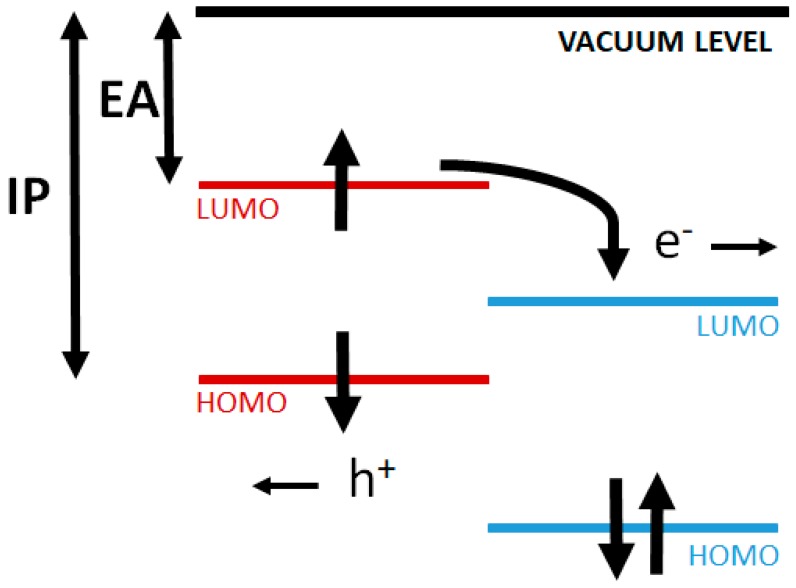
Charge separation of an exciton into a free electron/hole pair at a donor-acceptor interface.

The mechanism of electricity generation in OPVCs differs from that of inorganic cells, since no free charge carriers are generated. They comprise electron donor and electron acceptor materials: the donor absorbs the photons from the solar radiation, where excited states (or excitons) are created and confined [69]. An exciton is a bound state of an electron and an electron hole attracted to each other by electrostatic interactions, which can be separated into free electron-hole pairs by effective electric fields. The acceptor is the material acquiring the electrons from the dissociated electron-hole pairs. The charge separation of an exciton into a free electron/hole pair at the donor-acceptor interface is schematized in Figure 11.

Exciton dissociation is effective at the interface between materials with different electron affinity (EA) and ionization potential (IP) (Figure 11). The band gap in these materials is defined as the energy difference between the HOMO and LUMO levels. The open circuit voltage V_oc_ is the difference between the HOMO of the donor material and the LUMO of the acceptor material. The differences between the EA and the IP of both materials generate electrostatic forces at the interface which allow to generate stronger electric fields that can break the excitons more efficiently. Consequently, each exciton generates a load-free carrier that can be collected by the electrode of the material with higher EA, while the hole is accepted by the material with lower IP.

**Figure 12:**
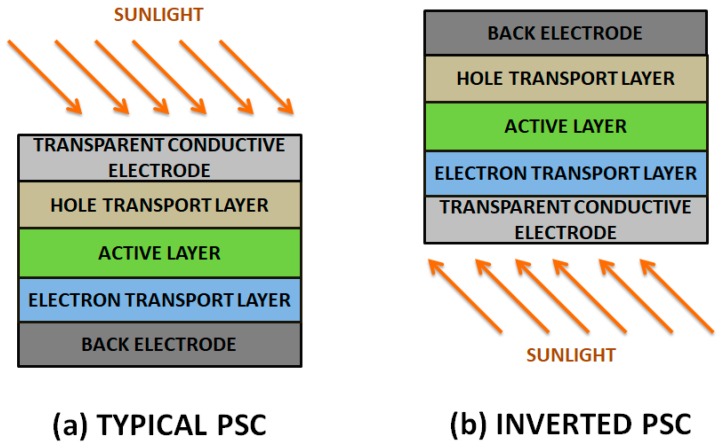
Schematic representation of a typical (**a**) and inverted (**b**) polymer solar cell (PSC). Taken from [69].

OPVCs can be further classified into two groups according to the chemical structure of the electron donor (p-type) semiconductor: polymer solar cells (PSCs) and small molecule solar cells. PSCs are usually made of an indium tin oxide (ITO) conductive glass covered by a polymeric hole transporting layer, an active layer, an electron transport layer and a low work function metal electrode like Al (Figure 12). In contrast, an inverted device comprises an ITO glass with a buffer layer as cathode and the anode is a high work-function metal like Ag or Au [70]. A high work-function metal (higher than that of the hole transport layer) and a low work-function metal (lower than that of the electron transport layer) are required in order to implement ohmic contacts, preventing the formation of Schottky junctions (which show rectifying behaviour, increasing the difficulty of current extraction, and decreasing device efficiency).

The order and chemical nature of the layers and the metal electrode strongly condition the performance of PSCs, and consequently their efficiency [71]. 

**Figure 13:**
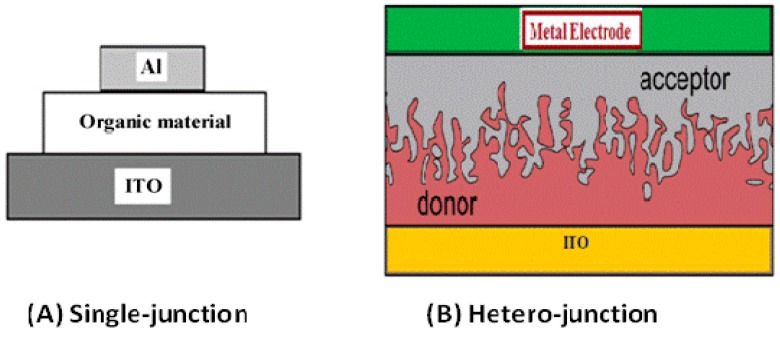
Schematic representation of (**A**) single-junction and (**B**) hetero-junction solar cells. Taken from [72].

PSCs can be configured as single-junction or hetero-junction, as schematized in Figure 13 [72]. The hetero-junction architecture consists in a sandwich connection of organic materials between two metallic conductors, typically ITO, separated by a metal coating such as Al, Mg, or Ca; the organic layer with the highest EA and IP value would be the acceptor, while the other would be the donor. A more efficient option is the use of bulk hetero-junctions (BHJ) [73], formed by blending an electron-rich conjugated polymer as donor and an electron-deficient fullerene as acceptor with a bicontinuous nanoscale interpenetrating network; since the area of the D-A interface is significantly increased, the exciton dissociation efficiency is improved compared to the other architectures. 

**Figure 14:**
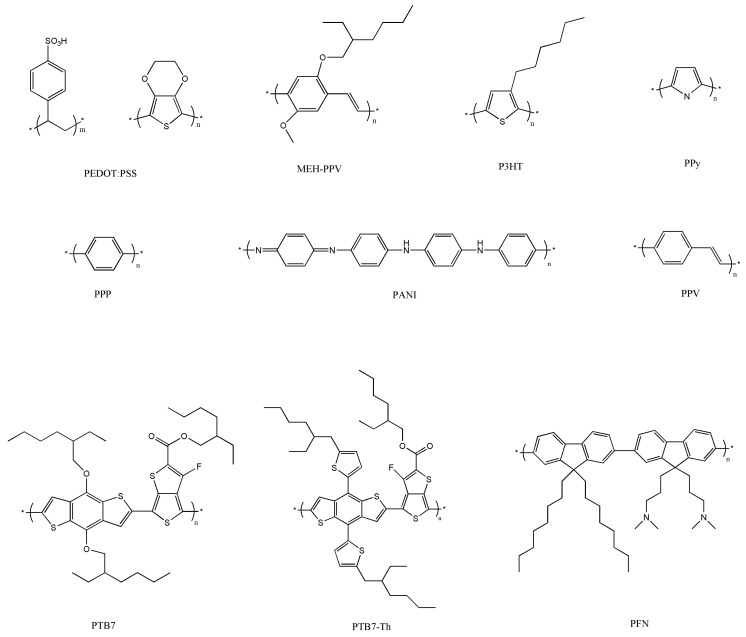
Chemical structure of conjugated polymers typically used in polymer solar cells. Taken from [69].

A very wide range of polymers have been used for the manufacture of PSCs [74,75,76]; the chemical structure of some of the most representative conjugated polymers employed in BHJ PSCs is shown in Figure 14. The alternating carbon–carbon double bonds are common to all of them, and are responsible for their electronic properties, low-energy optical transitions and high-electron affinities. Raw conjugated polymers are typically insulators, and become conductive via oxidation (*p*-doping) or reduction (*n*-doping). 

The developments in PSCs can be classified according to the layer in which the polymers are introduced. For the substrate design, little thickness and flexibility are desirable [77]. Thus, polyethylene terephthalate (PET) or polyethylene naphthalate (PEN) are frequently employed since they have good thermal and mechanical properties and good stability during solvent treatments [78]. 

Recently poly(3,4-ethylenedioxythiophene)-poly(styrene sulfonate) (PEDOT:PSS) has captured the interest of scientists due to its flexibility and processing capacity in solution [79]. In addition, PEDOT:PSS has good transparency, mechanical resistance, ability to transport holes, high work function, and good stability at room temperature. It has been applied as an anode, cathode, and as both electrodes simultaneously. In this regard, Hau et al. developed solution-processed functional devices, although they exhibited low efficiency [80]. On the other hand, PEDOT:PSS has several shortcomings including hygroscopic character and inhomogeneous electrical properties that result in low durability [81].

Poly(3-hexylthiophene) (P3HT), with a wide band gap of 1.9 eV, is also broadly used in PSCs owing to its superior electronic properties combined with its ability to self-assemble and to be easily dissolved in organic solvents. Polyaniline (PANI) is also very attractive owing to its low price, simple synthesis, and high environmental stability. In addition, it shows an acid/base doping response and its electrical properties can be tuned by changing its oxidation and protonation state [82]. On the other hand, other polymers display low solubility like poly(p-phenylene vinylene) (PPV), polypyrrole (PPy), and poly(p-phenylene) (PPP), which restricts their applicability. Further, PPV suffers from poor absorption and photodegradation [83]. 

PSCs suffer from different types of stresses, atmospheric exposure and electrical polarization [meter REF: Improving efficiency and stability of perovskite solar cells with photocurable fluoropolymers]. To improve the stability of the PSCs, fluoropolymeric coatings can be applied on both sides of the cell. Recently, fluoropolymers have been incorporated in PSCs due to their excellent optical and mechanical properties and their extraordinary stability against ultraviolet radiation. Thus, polytetrafluoroethylene (PTFE) has been used as the ITO buffer layer owing to its low chemical reactivity, high thermal stability, excellent moisture resistance, flexibility, light weight, and low cost [84]. 

Another aspect that should be considered for the development of electrodes is the work function, since it must decrease gradually for a correct charge transport. Currently, methods to reduce electrode values using ethoxylated polyethyleneimine (PEIE), and polyethylenimine (PEI) have been proposed [79].

**Figure 15:**
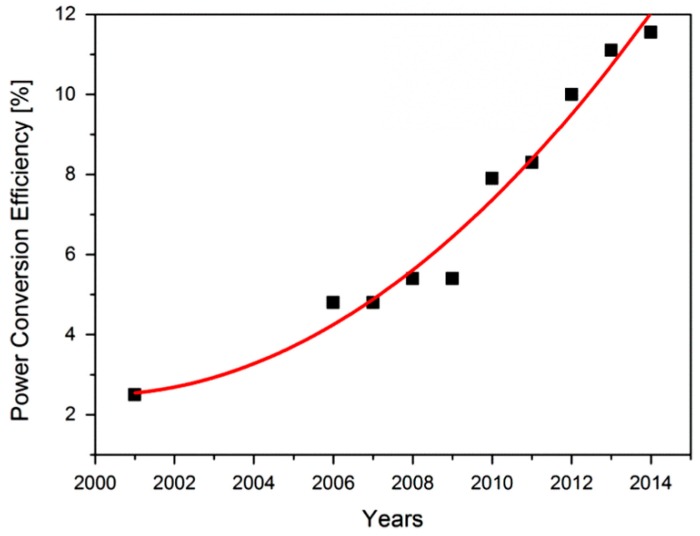
Power conversion efficiencies vs. time for OPVCs. Taken from [79].

OPVCs present lower efficiencies than cells based on inorganic compounds [73], owing to their technological and market maturity and other factors such as their wide band gap. The highest efficiencies reported range between 9.7–11.2% [13], and taking into account the increasing trend found within the last years, it is expected that they will improve further (Figure 15). The best laboratory performance in OPVCs (22.4%) has been attained with P3HT as donor material [73].

Overall, OPVCs display important advantages compared to previous generations including flexibility, lightweight, lower processing costs and less environmental impact [85,86]. In addition, the cell layers can be deposited via solution-based methods like spin-coating or printing, which enable device fabrication at a large scale at low temperatures, hence reducing the associated costs. PSCs can also be transparent at the expense of a lower efficiency, which is interesting for applications in windows, walls, flexible electronics, and so forth. Their main disadvantages are their low efficiencies and that are susceptible to photochemical degradation [83].

### 4.4. Perovskite Solar Cells

**Figure 16:**
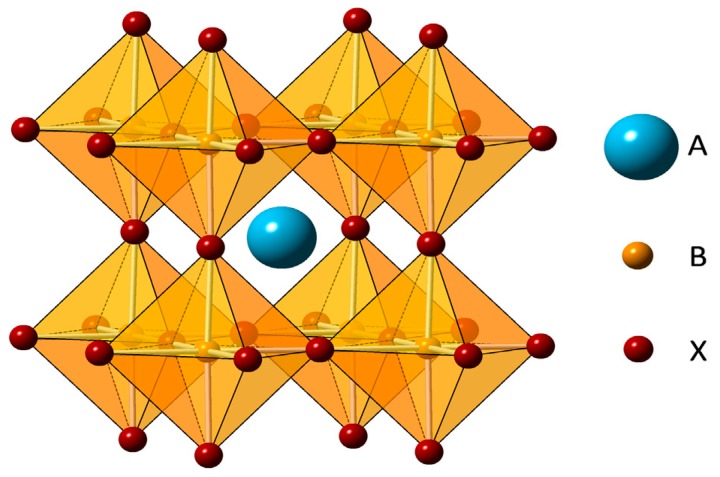
Perovskite structure: (A) monovalent cations, (B) lead, (X) iodine. Taken from [87].

Perovskite solar cells (PVSCs) include a perovskite structured compound (see Figure 16 [87]), most commonly a hybrid organic-inorganic lead or tin halide-based material, as the light-harvesting active layer, which is placed between the electron transport layer (ETL) (usually mesoporous material or flat TiO_2_) and the hole transport layer (HTL) [87]. In the standard device configuration, the front transparent electrode is a fluoride-doped tin oxide sheet (FTO), and the back electrode is a thermally-evaporated gold layer. 

PVSCs also have a D-A interface. However, unlike OPVCs, the excitons do not need to have a long lifetime since the absorption of photons practically results in the generation of free charge carriers [87]. This non-generation nature of excitons is critical for the device performance. The efficient generation of electrons and holes in a single stage is one of the main advantages of the PVSCs, since the energy losses due to the generation of excitons, their movement and their dissociation are avoided. In addition, the raw materials used and the fabrication methods (such as printing techniques) are both low cost [88]. They are simple to manufacture, and their high absorption coefficient allows to absorb the whole visible solar spectrum with ultrathin films (~500 nm) [89]. These characteristics make them ideal candidates to fabricate low cost, high efficiency, thin, lightweight, and flexible solar modules.

One the other hand, one of the main challenges for PVSCs is the short-term and long-term stability. Their instability is mostly associated to environmental moisture and oxygen, thermal influence, heating under and applied voltage, UV and visible lights and mechanical fragility [90,91,92].

In particular, the water-solubility of the absorber material makes the cells highly prone to fast degradation under moist environments. This degradation can be minimized by optimizing the constituent materials, the cell architecture, the interfaces and the environmental conditions during the fabrication stages. For instance, it has been reported that the encapsulation of the perovskite absorber within a carbon nanotube composite and an inert polymer matrix hinders the degradation in a humid atmosphere and at elevated temperatures [93]. 

Another key challenge for this type of cells is that their current-voltage curves show a hysteretic behaviour depending on the measurement conditions (i.e., scan direction, scan speed, light soaking, biasing). Several reasons have been proposed to explain such behaviour including ion movement, polarization, ferroelectric effects, filling of trap states, albeit the exact origin is not known yet [94].

In PVSCS, the separation of charges can take place either by the injection of the electrons photo-generated in the ETL or by the injection of the holes in the HTL. Further, the free electrons generated near the perovskite/HTL interface should diffuse through the full layer thickness before being extracted at the ETL/perovskite interface, which increases the possibilities of recombination [95]. Similar phenomenon occurs to the holes near the ETL/perovskite interface. Recent works have shown that both processes take place simultaneously [87].

Perovskite cells have improved their efficiency from an initial value of 3.8% in 2009 [43] to 22.13% in 2018 in single-junction architectures [96,97]. Current research focuses on improving efficiency, while trying to solve the aforementioned challenges of this type of cells [96]. It has been reported that solid-state perovskite cells exhibit higher efficiency than those with liquid electrolytes, therefore the use of solid electrolytes is recommended. The improvement of the efficiency is usually approached by means of the optimization of the perovskite composition, the deposition method applied, and the architecture of the device [96]:

- Composition: the highest efficiencies achieved (21.1–21.6%) have been obtained by mixing different cations to form the perovskite [98,99]. Further, band gaps are tunable and can be optimized for the solar spectrum by altering the halide content in the film (i.e., by mixing I and Br);
- Deposition method: a good interface between the different cell layers improves the efficiency. In this regard, a value of 19.7% has been reached when using DMF and DMSO as solvents during the spin-coating process [100]; and
- Cell architecture: this parameter strongly affects the final cell efficiency. For instance, the use of TiO_2_ with fullerene as ETL enabled the fabrication of a hysteresis-free device, with an efficiency of 19.1% [101]. Additional layers should also be considered, such as the anti-reflective coating, as well as the thickness and strength of each layer [102,103].

### 4.5. Multi-Junction Solar Cells

**Figure 17:**
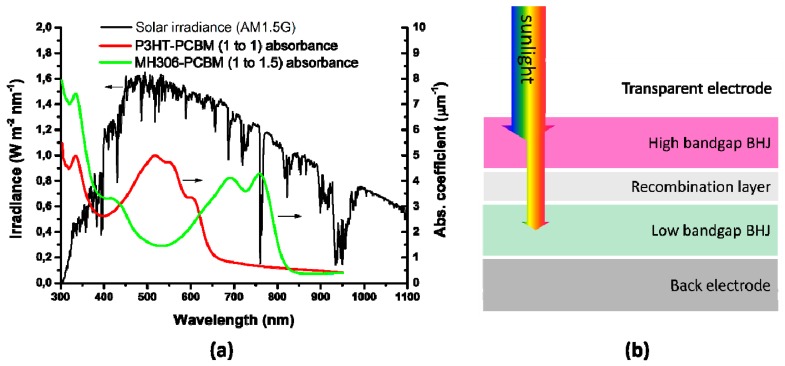
(**a**) Irradiance (black line) and absorption coefficient (red and green lines) vs. wavelength. (**b**) Schematic representation of a double-junction plastic solar cell. Taken from [104].

Multi-junction (MJ) solar cells comprise multiple p–n junctions made of different semiconductor materials, and each of them produces electric current in response to different wavelengths of light, thus increasing the conversion of incident sunlight into electrical energy and the device efficiency. The idea of using different materials with different band gap is proposed to take advantage of the maximum possible photons. The whole cell can be made of the same material or different ones, offering a wide range of design possibilities. A schematic representation of the general structure of a tandem cell with two junctions also known as double-junction cell, is shown in Figure 17 [104]. They typically include the following elements:

- A transparent electrode (i.e., TCO), which covers a larger band gap cell that will be the first to capture the radiation.
- A recombination layer, which is applied either as a tunnel connection or a TCO depending on whether a serial or a parallel connection of the cells is required.
- A cell with lower band gap than the previous one.
- A back contact.

**Figure 18:**
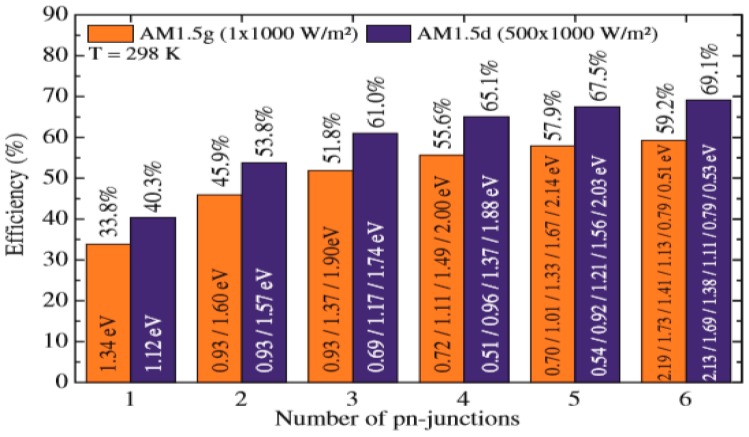
Efficiencies for MJ cells as a function of the number of junctions. Taken from [105].

In general, there are two types of designs: the monolithically integrated tandem and the mechanically stacked tandem. Nevertheless, it should be noted that the mechanical stacking technology is difficult to scale up and relatively expensive nowadays. The manufacture of multi-junction devices is challenging since the extraction of currents is not trivial. The theoretical efficiencies for MJ cells with different number of unions are plotted in Figure 18.

Clearly, as the number of junctions increases, the efficiency raises. Nevertheless, a high number of junctions makes the choice of the different layers difficult, and increases the complexity of the device; therefore, the optimum balance between these three parameters has to be investigated.

**Figure 19:**
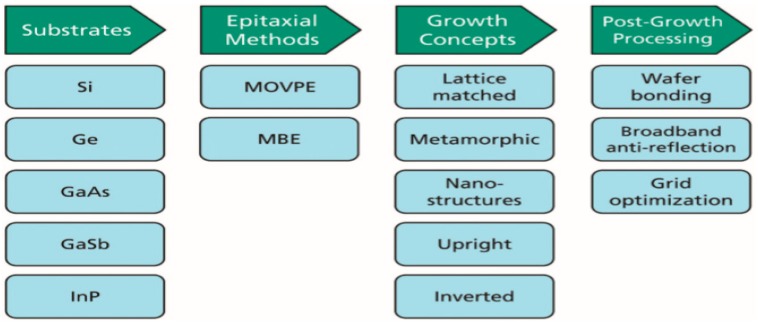
Technological tools and processes that are used to design new high-efficiency III-V multi-junction solar cells. Taken from [105].

This type of cells appears to be the only realistic option to achieve extremely high efficiencies, and the monolithic structure will lead to the best results [105]. However, the manufacturing of large surface cells requires expensive production processes, thus the combined use of multiple smaller cells to cover the same area seems to be economically more viable nowadays. An alternative to increase efficiency is the combination of these smaller cells with concentration technologies, which concentrate hundreds or thousands of times the sunlight, leading to higher energy production [105]. Currently, 40% efficiency has been surpassed with multi-junction cells [106,107,108,109], and they are expected to even surpass 50% [110], although significant research is carried out in different directions, as depicted in Figure 19.

As can be observed in the figure, the substrates are often based on 1GEN and 2GEN compounds. For instance, an efficiency of 35.8% has been achieved with an InGaP/GaAs/InGaAs cell on a Ge substrate, and it is expected that with further improvements, efficiencies higher than 50% could be attained, hence becoming a promising alternative for terrestrial and space applications [111]. Several growth methods can be applied, and allow the synthesis of different structures focused on different applications. A well-studied alternative over recent years is the metamorphic growth, which is based on the progressive monolithic growth of materials with gradually higher lattice constants [107,112,113]. This growth would likely reduce the price of substrates used in cells based on chemical compounds of the III–V groups [105]. Currently, efficiencies close to 40% have been reached under sunlight concentration, although theoretical calculations indicate higher values.

Similarly to OPSCs, inverted cell growth is feasible, as well as its combination with another techniques such as metamorphic growth to achieve better efficiencies [105]. One of the advantages of the inverted growth is that the growth of the buffer layer is delayed, hence the potential dislocations due to the transfer of the lattice constant do not affect the cells located on top. Another advantage is that the material employed to fabricate the bottom cell can be more flexible depending on whether an epitaxial growth is performed with or without Ge. In addition, the substrate can be recycled to manufacture several cells, which would reduce the large-scale production costs. By applying this inverted metamorphic growth, cells with efficiencies between 40–44.4% have been developed [114].

Overall, albeit MJ solar panels are highly efficient, are more expensive than other technologies, and this implies different applications: MJ solar cells are preferred in space whilst c-Si solar cells are better for terrestrial applications. In order to extend the use of MJ cells, large-area, cost-effective, and highly reproducible fabrication processes need to be developed. Nevertheless, MJ solar cells do have the potential to have an important penetration in terrestrial applications in concentrator systems.

## 5. Fourth-Generation Photovoltaic Solar Cells

**Figure 20:**
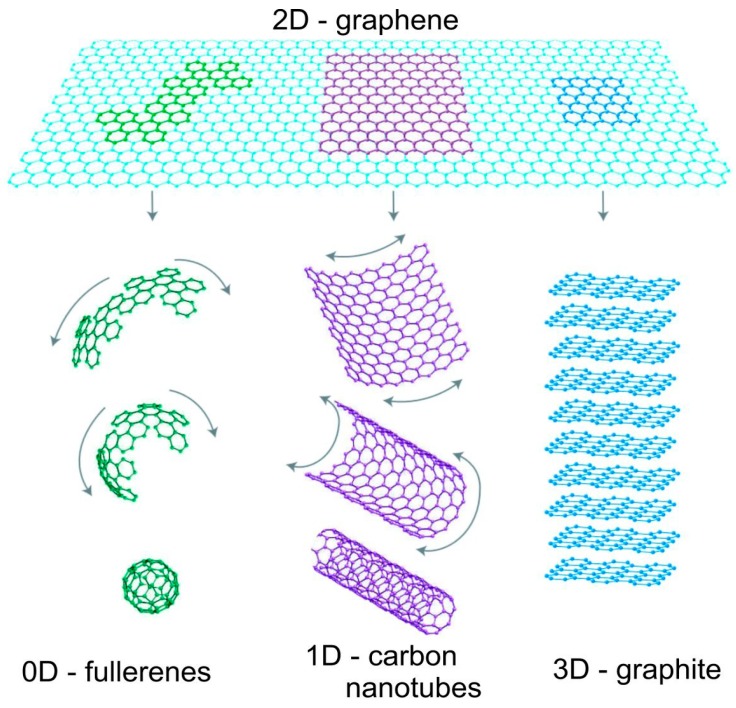
Different arrangements of honeycomb like carbon atoms depending on their dimensionality. Taken from [115].

The 4GEN combines the low cost/flexibility of polymer thin-films with the good stability of nanomaterials like metallic nanoparticles, metal oxides, carbon nanotubes, graphene, and its derivatives. These architectures will maintain the advantage of solution processable devices, hence cheap manufacture, but also incorporate nanomaterials to improve the charge dissociation and charge transport within the cells. In particular, special emphasis is placed on graphene (G), which has become the nanomaterial with the highest scientific and technological expectations. Currently, some researchers consider G as the fundamental unit of graphitic structures [115]; the different arrangements of honeycomb like carbon atoms lead to different allotropes with different dimensionalities, as depicted in Figure 20: OD fullerenes, 1D carbon nanotubes, 2D graphene, and 3D graphite. 

### 5.1. Graphene and Its Derivatives

G is a two-dimensional material composed of carbon atoms that form an atomically-thick layer with a honeycomb lattice structure (Figure 20). The carbon atoms are linked by covalent bonds in the same layer and by Van der Walls forces between layers. Each layer presents a flat sheet appearance although, due to the carbon hybridization and the possible imperfections and impurities, it presents small undulations. The sp^2^ hybridization of carbon atoms also determines that the free electron of the 2px orbital can form π bonds with other atoms. This chemical configuration gives the material exceptional chemical, electronic and mechanical properties. G is an exceptional electrical and thermal conductor: its electrical conductivity is superior to that of Cu or Si [116], and it has such a high thermal conductivity (G: 3.000–5.000 W·m^−1^·K^−1^ vs. Cu: 400 W·m^−1^·K^−1^ [117,118]) that it is one of the best thermal conductors. G also presents very high electron mobility (15,000 cm^2^/V s) and very large specific surface area (SSA ≈ 2.630 m^2^/g [118]). In addition, G is one of the strongest materials on earth, with an elastic modulus close to 1 TPa, a tensile strength of 130 GPa and a breaking strength of ∼40 N/m [118,119]. The combination of these exceptional properties make G an excellent candidate for application in photovoltaic cells. Further, G sheets are flexible and chemically inert, which leads to a double role application: as an electrode and as a protective layer. Nonetheless, some issues need to be solved. G is almost transparent since it just absorbs 2.3% of the radiation [118]; this can be a problem for solar cell applications, since it is not able to capture the photons, but can be solved by means of doping.

G can be obtained by different production methods. The mechanical exfoliation of the graphite allows to obtain clean surfaces of G, which present a high mobility of load carriers [120,121]. It can also be obtained by chemical exfoliation of graphite, either by means of a dispersion with metal ions [118,122], or a simple sonication process followed by a dispersion in an organic solvent. Another approach is the epitaxial growth of G on SiC [118,121] by vacuum graphitization (thermal treatment of SiC at ~1300 °C under vacuum). Although the G obtained by this method has good properties and can be molded by lithography methods, the applicability to large-scale production is limited due to the high cost of monocrystalline SiC sheets [118]. In addition, a previous work has shown that the properties of G depend on the effects of the interface with the substrate sheet [121]; in this regard, the use of Ru has been investigated, and epitaxial G was successfully grown [121]. Chemical vapour deposition (CVD) is the most widely used since allows obtaining large quantities of G in a simple and fast way [123]. G grows on a substrate (usually Ni, Cu, Pd, Pt, or [121,124]) as a C gas steam (usually methane) flows through the chamber; G can be doped by adding other gases during the process [118]. The CVD method enables to obtain G with few structural defects at a large-scale. Some drawbacks of this method are the control of the G film thickness and the elevated cost of the substrates.

**Figure 21:**
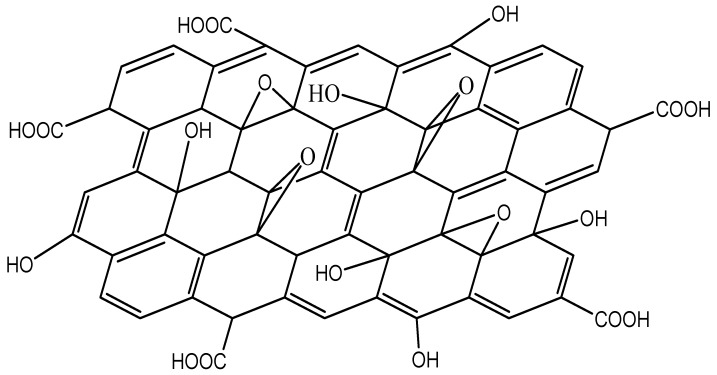
Chemical structure of graphene oxide (GO).

Graphene oxide (GO) is a modified form of G that incorporates carboxylic groups at the edges of the sheet, as well as epoxide and hydroxyl groups within the basal plane [125]. The chemical structure of GO is schematized in Figure 21. Some properties of GO are different from those of G. The anchored groups and lattice defects change the electronic structure, hence it presents considerably lower electron mobility and electrical conductivity, being usually insulating (sheet resistance of 1012 Ω/sq). Conversely, it has larger surface area and better ability to retain compounds in the interlaminar space. It can be processed in aqueous solutions, has amphiphilic character and surface functionalization capability, it is versatile, biocompatible and can form stable aqueous colloids to enable the assembly of macroscopic structures, which is decisive for large-scale applications [126]. Additionally, it can be deposited on a substrate and later converted into a conductor, henceforth being a perfect candidate to be used in solar cells.

GO can be synthesized from graphite by means of a chemical exfoliation of graphite oxide [127], or from GO using four main methods [121,126]: Staudenmaier, Hofmann, Brodie, and Hummers. The most usually employed is the Hummers’ method, which consists in an oxidation process using KMnO_4_, NaNO_3_, and H_2_SO_4_ [128].

Reduced graphene oxide (rGO) can be obtained by heat treatment or by chemical or electrical reduction of GO to eliminate some functional groups [125,129]. Different reagents including hydrazine monohydrate (N_2_H_4_·H_2_O), NaBH4, hydroiodic acid (HI) and urea have been employed for the chemical reduction of GO into rGO [130,131]. rGO has better electrical properties than GO [126], owing to its lower degree of functionalization, hence it is also a good candidate for solar cell applications. 

### 5.2. Graphene Applications Tendencies

**Figure 22:**
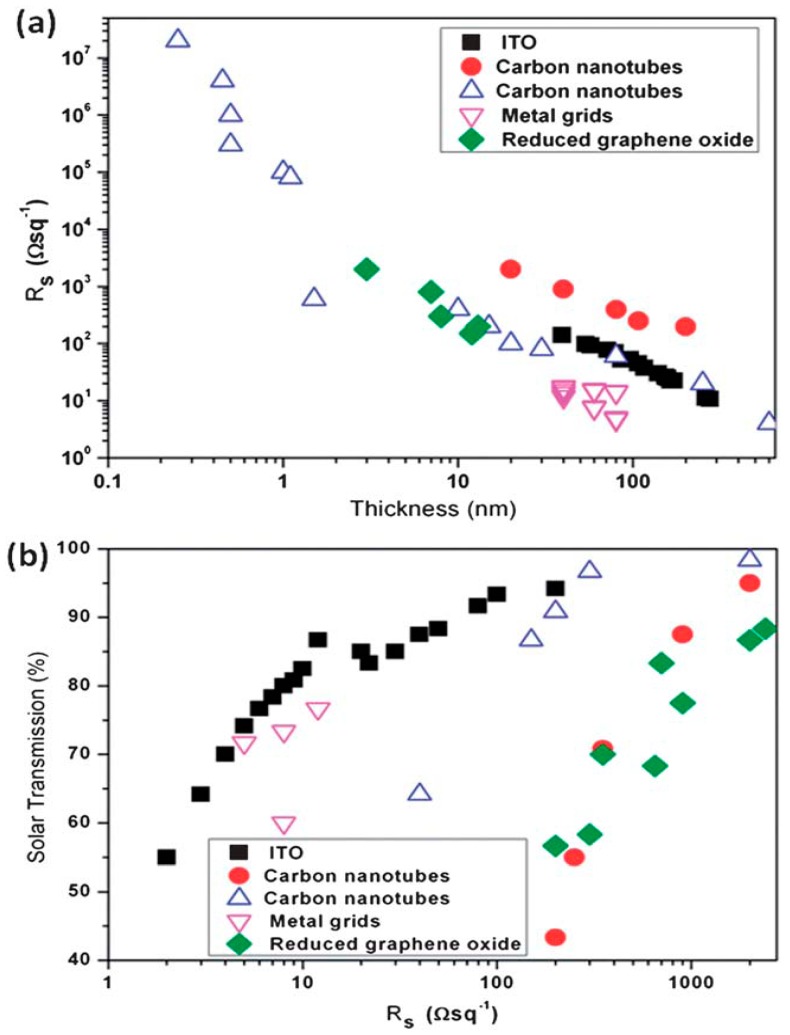
(**a**) Dependence of the sheet resistance on the film thickness and (**b**) dependence of solar transmission over the whole spectrum on the sheet resistance for ITO, carbon nanotubes, metal grids, and reduced graphene oxide electrodes. Taken from [5].

**Figure 23:**
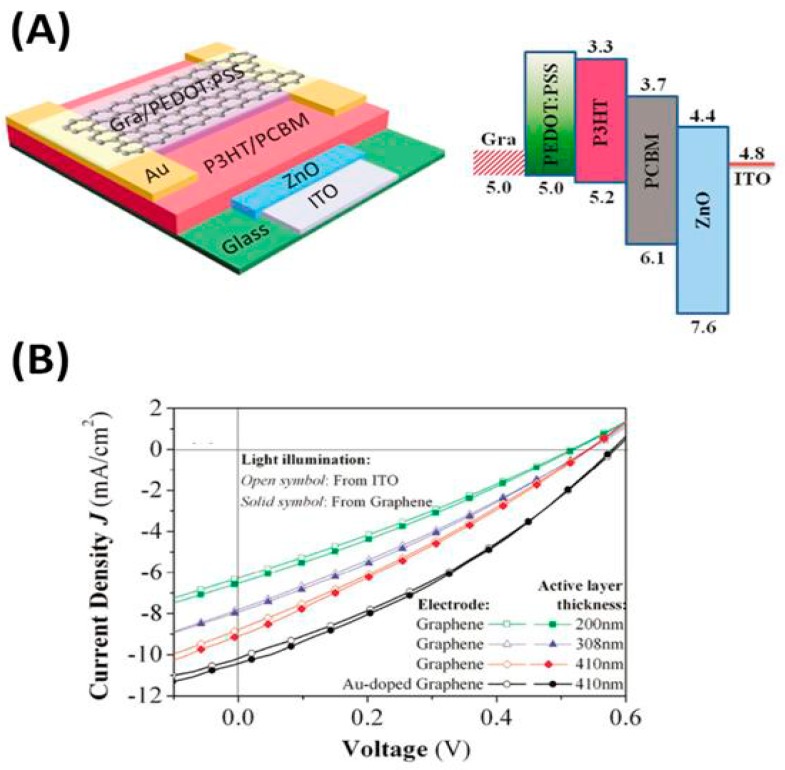
(**A**) Schematic representation and band structure of a PSC with the structure Glass/ITO/ ZnO/P3HT:PCBM/Au/PEDOT:PSS/G; (**B**) J–V characteristics measured from two sides of the PSC with G top electrode and different active layer thicknesses. Reproduced with permission from [137].

G is a promising material for energy-related applications, both in inorganic and organic cells, hybrids cells and DSSCs [118]. It is mainly used as an additional layer in functional cells of previous generations, since it allows to obtain cells with improved properties. In particular, it has been mostly used in transparent conductive electrode (TCEs), active layers and interfacial layers, that is, the hole transporting layer and electron transporting layer between the active layer and the anode/or cathode, respectively [123,132]:

(a) G as a transparent conductive electrode:

G and its derivatives have been used as TCEs owing to their good flexibility and transparency, to replace conventional ITO electrodes in PSCs [132,133]. The efficiency of a PSC with a transparent G electrode is 3.98% compared to 3.68% for the same PSC with an ITO electrode [123], which leaves a small margin of improvement in the cell manufacturing. Modern micro fabrication techniques, using roll-to-roll printing techniques [134], enable the synthesis of single graphene sheets over a large area without defects or structural kinks, thus increasing conductivity. Nonetheless, in order to reach the sheet resistance desired for a TCE (15 Ω/sq), about 20 G monolayers need to be stacked, which, in turn, can result in a strong fall in light transmission, close to 50% (Figure 22). To overcome such problems, the surface of G can be chemically functionalized or combined with conducting polymers via physical interactions [127]. For instance, a PEDOT:PSS layer coated onto a G-based electrode fills the gaps between the G sheets, thus improving conductivity, and acts as an energy step from the electrode to the active materials. Further, a graphene/CNT composite as electrode has better performance than the individual components [135]. The best approach is to integrate low amount of G within a transparent conducting host matrix, thus increasing transparency without reducing conductivity [136]. Semitransparent PSCs with a CVD-grown monolayer G doped with Au nanoparticles and PEDOT:PSS as top TCE and ITO as the bottom electrode have been developed (see Figure 23 [137]). The use of a doped G electrode strongly increased the conductivity (~400%) compared to raw G and led to a better transmittance. A very similar device with multilayer doped G as TCE on polyimide (PI) substrates was also manufactured [138], which exhibited outstanding flexibility and stability. Further, air was not able to diffuse across the interlayer spacing of the G layers, thus providing an excellent packaging effect. Multilayer G can act as a barrier and protect PSCs from air oxidation, making easier the cell fabrication process and diminishing the associated costs.

(b) G in active layers 

Most OPSCs are based on the BHJ architecture, in which the ideal active layer consists in a bicontinuous interpenetrating network of donor and acceptor at the nanometer scale with maximum interfacial area. The efficiency enhancement in BHJ solar cells compared to other architectures is attributed to the more efficient exciton dissociation enabled by the larger heterojunction interface and the better charge carrier collection due to the formation of an interpenetrating donor-acceptor network [139]. In this regard, G shows great potential to be used in active layers owing to its 2D structure and large specific surface area that would promote the development of an interpenetrating donor-acceptor network at the nanoscale.

**Figure 24:**
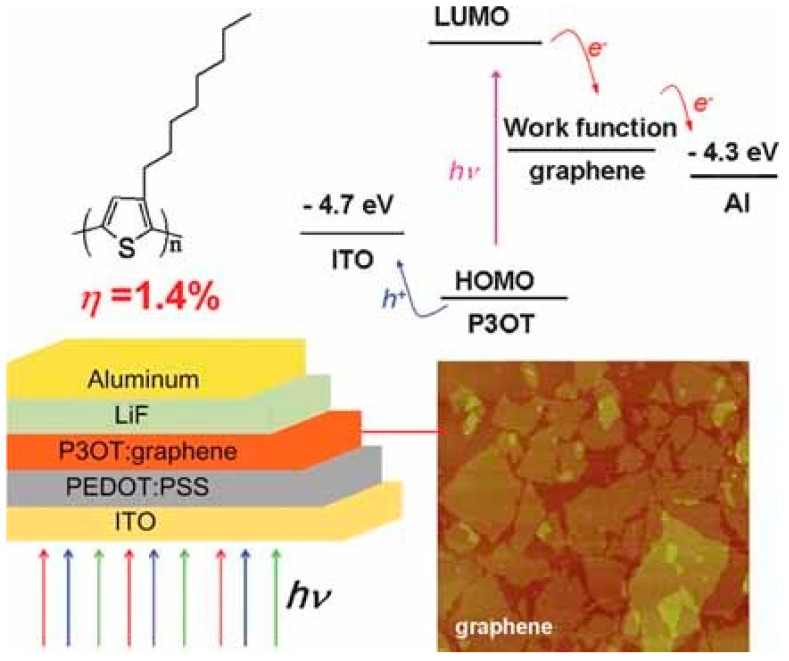
Schematic representation of a PSC with (P3OT)/functionalized G as the active layer. Taken from [142].

The first use of solution-processable functionalized G as an electron-acceptor material in PSCs was reported by in 2008 by Liu and coworkers (Figure 24 [140]). The functionalized G was prepared in two stages: firstly, GO was synthesized using a modified Hummer’s method, and then it was reacted with phenyl isocyanate, leading to a hydrophobic material that could be dispersed in organic solvents used for PVCs applications. Then, a composite film of poly(3-octylthiophene) (P3OT) as a donor and the functionalized G as acceptor was prepared by spin coating. The π-π interactions between P3OT and functionalized G made this composite work well as active layer in BHJ devices, albeit the efficiency was low (~1.4% for a G concentration of 5 wt % optimized by an annealing process at 160 °C for 20 min). The annealing process removes some functional groups, thus improving the charge transport, and increases the crystallinity of the donor matrix, consequently enhancing the properties of the optical active layer. 

A P3HT/solution processable G/functionalized multiwalled carbon nanotubes (f-MWCNTs) composite has also been used as an active layer in PSCs, where G acted as electron acceptor, P3HT as donor and the f-MWCNTs provided percolation paths of holes [141]. However, the efficiency of the resulting device was also low, about 1.05%. Improved efficiency is expected by changing the G content and processing conditions, since theoretical simulations envisage efficiencies higher than 12% for G-based PSCs.

(c) G and its derivatives as a hole transport layer (HTL):

The most common commercially available material used as HTL in organic electronics is PEDOT:PSS. However, the highly-acidic and hygroscopic nature of the polymer mixture, its non-homogeneous electrical properties and low stability over time has initiated the search for air-stable effective replacement materials. 

G is an interesting material to be used as HTL [123], particularly in its oxidized form, since GO has a lower work function compared to pristine G, making it better for hole injection. A GO/PEDOT:PSS composite has been used as HTL in a BHJ PSC, resulting in an efficiency of 4.28%, higher than those of devices with only GO (2.77%) or PEDOT:PSS (3.57%), as well as in better reproducibility and stability over time. The enhanced performance is due to the well matched work function between GO and PEDOT:PSS that increases the mobility of charge carriers. [143]. In addition, GO establishes a band-gap of 3.6 eV, making it possible to block electrons, and avoids the acid corrosion problems in the ITO layer caused by PEDOT:PSS, thus increasing the useful life of the cell. However, the homogeneity of the layer must be ensured and the GO thickness maintained between 1–3 nm to optimize its behaviour. On the other hand, partial-reduction of GO in order to rise conductivity both in vertical and lateral directions has also been tested, leading to devices with efficiencies surpassing that of PEDOT:PSS [81]. Recently, a BHJ PSC with a HTL made of a PEDOT:PSS/GO composite (1:1 *w*/*w*) was developed [144], resulting in an efficiency of 5.22%. The improved performance was ascribed to the reduction of the HOMO-LUMO gap by GO and the impedance reduction, thus improving the charge transport.

(d) G and its derivatives as an electron transport layer (ETL):

There are only a few ETL materials, the most widely used being highly reactive alkali earth metals like calcium or magnesium (with low work functions), or lithium fluoride. These materials have to be evaporated, requiring high vacuum and melting of metals to high temperatures for deposition. Owing to the energy-band structure of G that results in efficient charge transport and its superior mechanical properties, G and its derivatives are postulated as suitable materials as ETL [123]. In particular, the use of GO will enable the full device fabrication via roll-to-roll processes. Further, GO can tune the energy levels through chemical modification, allowing to function for electron injection instead of hole injection. For instance, the esterification of the COOH groups of GO with Cs_2_CO_3_ in water reduced its work function from 4.7 eV to 4.0 eV. The resulting device, that used the modified GO as both ETL and HTL, showed better performance than the reference with Cs_2_CO_3_ [145].

**Figure 25:**
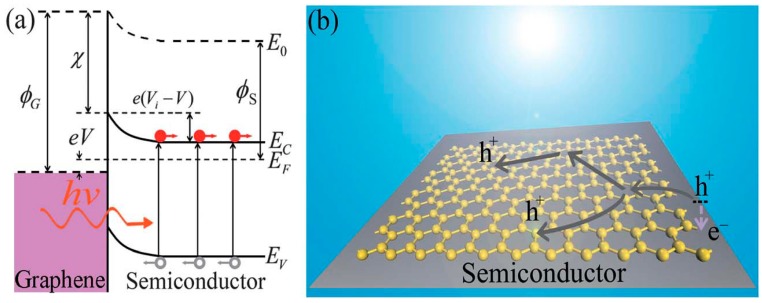
(**a**) Energy diagram of a semiconductor/G Schottky junction solar cell under illumination. *Ø*_G_ and *Ø*_S_ are the work functions of graphene and the semiconductor, respectively. eV_i_ is the built-in potential, V is the output voltage, E_C_, E_V_, and E_F_ correspond to the conduction band edge, valence band edge, and Fermi level of semiconductor, respectively, and E_0_ is the vacuum level. (**b**) Schematic illustration of G-based Schottky junction solar cell. Photogenerated holes (h+) and electrons (e−) are driven towards the G and semiconductor layers, respectively. Taken from [146].

The Schottky junction, formed by contacting a metal with a doped semiconductor, is a potential structure in solar cells [146]. It presents the advantages of material universality, performance stability inexpensiveness and easy of fabrication compared to the p–n junction. However, in this type of cell, the metal layer would absorb most of the solar radiation and consequently limit the energy conversion efficiency. To solve this drawback, ITO film has been used to replace the metal film, albeit this results in high production costs and restricts its use for flexible devices. Since G presents very high conductivity and a near-zero band-gap, G/n-type semiconductor heterojunction can be used as a metal/semiconductor Schottky junction. The mechanism of such solar cell can be explained considering the energy band diagram (Figure 25). Owing to the work function difference between G and the semiconductor, a built-in potential is formed in the semiconductor near the interface. Under light, the photogenerated holes and electrons are driven towards the Schottky electrode (G) and semiconductor layer, respectively, by the built-in electric field. When the solar cell is short-circuited, the extracted photogenerated carries will generate a short-circuited current. Compared to traditional Schottky junction solar cells, more light can go through the Schottky G electrode and excite electron–hole pairs in the semiconductor, leading to increased efficiency.

Different types of G-based Schottky junctions have been reported, including G-on-silicon ones [147,148]. These devices include a silicon square window patterned by etching off a SiO_2_ layer on an n-Si wafer. Then, a G sheet contacts with the Si window to form a Schottky junction; the G layer also acts as anti-reflection coating, reducing reflection, and the device efficiency was ~1.5%. A G-on-silicon Schottky junction cell with a PCE of 8.6% was developed by using G doped with bis(trifluoromethanesulfonylamide) [(CF_3_SO_2_)_2_NH] (TFSA) [149]. Overall, the G-based Schottky junction cells reduce the cost of the traditional Si-based solar cells, their fabrication processes are easy and compatible with Si microelectronic technology, and can be used in flexible devices.

### 5.3. Current Research on G Applications

Current research on G and its derivatives is focused on two main aspects: (a) the replacement of materials used in previous cells to improve some properties or reduce costs; and (b) the incorporation into previously studied materials, leading to enhanced cell performance. 

#### 5.3.1. Replacement of Other Materials

The replacement of some materials by G is motivated by its excellent properties that can improve the cell operation and its economic competitiveness; however, the performance improvement can lead to a detriment in other properties. For instance, the use of a thin G sheet as a substitute for Pt to manufacture conducting electrodes in DSSCs with PEDOT:PSS decreases the efficiency from 4.39% to 3.95%, albeit the cell durability is improved, and the efficiency is maintained between 2.37–3.23% after 100 cycles of flexion, values significantly higher than the one obtained using Pt (2.08%) [118,150].

Also in DSSCs, liquid electrodes have been replaced by rGO with electrolyte gels (such as polyethylene oxide (PEO) doped with gamma-butyrolactone, LiI and I_2_) to improve efficiency [151]. In the case of PEO, efficiency values of 5.7% were achieved owing to the faster diffusion of the I_3_^−^ ions and other ionic species and the plasticizing effect of the carbon sheets.

In the field of inorganic cells, G can also be used to improve designs; for instance, a perovskite cell used an ETL based on G/polymer composite (a mesoporous layer of rGO/PANI deposited on an ITO substrate) to solve thermal and chemical stability issues [152]. This type of combination allows achieving an efficiency of 13.8% under solar lighting. Further, some authors proposed the substitution of ITO for CVD-grown G [153], or its use as a transparent anti-reflective coating [154]. Thus, a G/PEDOT:PSS layer was deposited by spray coating as a cathode in an inverted PSC, albeit the efficiency dropped slightly compared to the device with ITO, which indicates that an optimization is required. In addition, it was found that ITO worked better in the long wavelength region, while GO worked better with short wavelengths [153].

#### 5.3.2. Incorporation into Other Materials

Some authors proposed to use G sheets coated with ZnO nanocrystals for infrared radiation capture, since it corresponds to the 40% of the incident solar radiation [154]. In addition, if randomly-distributed gold nanoparticles were used as dopants and the G thickness was maintained at 20 nm, the overall yield would increase to 8.94%.

The use of G sheets as a dopant has also been studied; in particular, it has been added to PEDOT:PSS solutions in isopropyl alcohol to obtain a material with a conductivity 10 times higher, and with a transparency comparable to ITO [155]. By dissolving PEDOT:PSS in the alcohol, the conductivity is improved due to charges excess, but the roughness worsens and the surface defects are increased. Upon addition of the G sheets, the mechanical strength and integrity of the whole layer are improved.

The addition of rGO to PEDOT:PSS as HTL in hybrid Si-organic cells (HSCs) has also been studied [156]. The combination of rGO with PEDOT not only provides new routes for the charge transport in the HTL, that improve the mobility and efficiency in the collection of the carriers, but also suppresses the recombination of the electrons at the interface. Moreover, the rGO acts as an antireflection coating and reduces the reflectance of PEDOT, thus improving the performance of the cells. Overall, the addition of rGO improved the electric conductivity of the PEDOT:PSS by 35%, and with a concentration of 2 mg/mL, an efficiency of 11.95% was attained. However, an excess of rGO can induce a faster cell deterioration due to the formation of additional defects.

G, GO functionalized with Li or rGO can be added to increase efficiency in perovskite cells. The idea is to either add or to replace the TiO_2_ by one of these nanomaterials, leading to an efficiency in the case of Li-GO of 11.8% [153]. The improvement is attributed to the presence of G that reduces the recombinations of the electron-holes [118]. Further, rGO/PEDOT:PSS composite can be used as a HTL layer in this type of cells [157,158]. The use of rGO hardly changes the surface roughness, thereby favouring the deposition of the perovskite absorber and improving the interface. With an optimal rGO:PEDOT:PSS 1:1 ratio, a maximum efficiency of 10.3% was obtained [157,158]. In addition, the rGO can be used as a heat sink to extend the useful life by avoiding mechanical stresses caused by heat.

Other authors added rGO to PMMA-based cells to form rGO/PMMA nanocomposites [159], and a maximum efficiency of 25% was obtained for an optimum G concentration of 1.0 wt %.

### 5.4. Carbon Nanotubes 

Carbon nanotubes (CNTs) are allotropes of carbon discovered by Iijima in 1991 that present a tubular structure formed by curling G sheets [160]. There are two main types of CNTs: single walled CNTs (SWCNTs), which consist of a single tube of graphene, and multi-walled CNTs (MWCNTs) that are composed of several concentric tubes of G. SWCNTs can be further classified into metallic and low band gap semiconductors, while MWCNTs are metallic in nature [161]. Different techniques have been developed to synthesize CNTs, including arc discharge, laser ablation, high-pressure carbon monoxide disproportionation and CVD processes [162]. CNTs have unique electronic, chemical and mechanical properties that make them interesting materials for a range of applications [163]. They present very high length-to-diameter ratio, in the range of 103–105, and are among the strongest and stiffest materials known, with an elastic modulus close to 1 TPa [164]. They also display very high electrical conductivity; metallic nanotubes can carry an electric current density of 4 × 10^9^ A/cm^2^, which is more than 1000 times greater than those of metals such as Cu [165,166].

Further, they are very good thermal conductors along the tube, showing ballistic conduction at room temperature. SWCNTs present a thermal conductivity of ~3500 W·m^−1^·K^−1^ [167], significantly higher than that of Cu (385 W·m^−1^·K^−1^), and possess very high thermal stability under both air and inert conditions. 

Mainly, CNTs can be applied in PVSCs in three ways: as TCEs, as transport layer or in active layers. 

#### 5.4.1. CNTs as Transparent Conductive Electrodes

CNTs have a low percolation threshold, hence just a low concentration is needed to attain a low sheet resistance suitable for the development of TCEs. However, the contact resistance between individual nanotubes is very high, hence CNT films alone need to be more than 100 nm thick to attain a sheet resistance [168]. Even so, CNT films manufactured by an inexpensive screen printing technique on glass and on elastic polymer substrates displayed high optical transmittance, and were tested as TCEs in a standard silicon cell. The best efficiency obtained (8.6%) was lower than that of a cell with conventional ITO, owing to the elevated resistance among CNTs [169].

To overcome the issue of conduction between individual nanotubes, researchers have looked at adding functionality to the CNT surface that can act as a conducting bridge.

#### 5.4.2. CNTs in Transport Layers

CNTs can be used as HTL, since have excellent energy-level matching for injecting holes and are environmentally-friendly materials. Further, they present high thermal and chemical stability, making them perfect candidates to replace conventional PEDOT:PSS HTLs. Owing to the possibility to functionalize CNTs with a variety of functional moieties, the work function and affinities can be modified for either electron or hole transport/injection. Achieving a good dispersion of CNTs or G within the polymer matrix is the key to fabricate composites with enhanced mechanical, electrical, and thermal properties [161]. However, raw CNTs have a strong tendency to aggregate and form bundles, and G sheets to remain stacked together via π-π stacking interactions, hence new approaches to homogeneously disperse these nanomaterials and keep a large specific surface area when blended with polymers have been reported including the use of surfactants [170] or polymer functionalization by means of covalent [171] and non-covalent approaches [172]. Covalent functionalization takes place via chemical modification of the CNT sidewall, either by acid treatments that generate hydroxyl and carboxyl groups, or via anchoring of small molecules or polymeric chains [173]. The covalent functionalization damages the nanotube sidewalls, while the non-covalent functionalization maintains the integrity of the nanotubes since the polymers interact via van der Waals forces and π-π stacking interactions [174].

**Figure 26:**
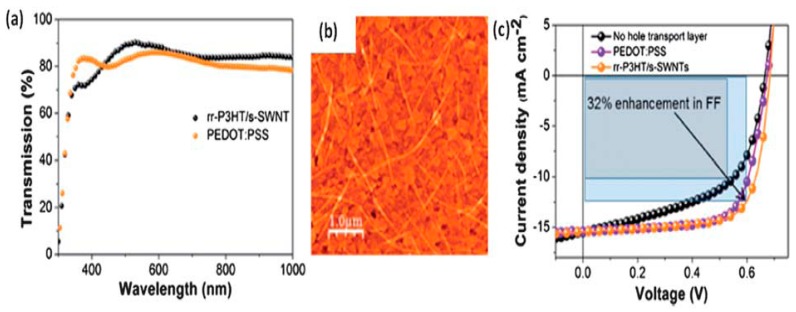
(**a**) Optical transmission, (**b**) atomic force micrographs, and (**c**) J–V characteristics of P3HT/SCWNT composites as hole extraction layers. Taken from [127].

Using non-covalent functionalization of P3HT onto SWCNTs, a thin film with transparency equivalent to PEDOT:PSS was obtained, leading to a PSC with higher fill factor and efficiency than the cell with PEDOT:PSS as HTL (Figure 26 [127]).

One of the advantages of the carbon-based nanomaterials is the ability to control their energy levels by chemical modification, allowing them to act as ETL instead of HTL. The first trial step was reported by Kymakis and Amaratunga [175] who used a P3OT/SCWNT composite as ETL. The addition of SCWNTs led to a strong increase in efficiency attributed to the enhanced exciton dissociation at the polymer-nanotube interface. Carbon nanotubes were tip sonicated in the solvent prior to polymer addition, in order to improve their dispersion. Similarly, Pradhan et al. [176] functionalized MWCNTs to achieve a better dispersion in a P3HT matrix and used the resulting composite as ETL. Improved efficiency was also attained, ascribed to the hole transporting nature of the CNTs which were expected to act either as high mobility charge pathways or as bridging sites for better percolation in the polymer phase. 

There is controversy regarding the actual role of CNTs. While certain groups claim them to be a hole transporter [177,178,179] others assert that they are electron transporters [175,180,181]. Furthermore, there exists opposing proofs for the preferred charge transportation via nanotubes under the same mechanisms. Understanding their charge transport properties is crucial prior to incorporating them as donors or acceptors in PSCs. 

#### 5.4.3. CNTs in Active Layers

CNTs—in particular, MWCNTs—have been used in active layers of PSCs. One of the major reasons for the use of MWCNTs instead of SWCNTs is their more uniform electronic properties. Additionally, controlled doping of MWCNTs by suitable treatments improves the cell performance. N- or B-doped MWCNTs uniformly dispersed in the active layer of a bulk-heterojunction solar cell consisting of P3HT and a fullerene derivative, PC71BM, selectively enhanced electron or hole transport and eventually aid carrier collection. The doping using thermal treatments with an ammonia/argon gas introduces nitrogen ions into the wall, lowering their work function from 4.6 eV for the pristine MWCNTs to 4.4 eV; in contrast, B is added to make the CNTs more p-type. In particular, the addition of 1.0 wt % B-doped MWCNTs resulted in balanced electron and hole transport, leading to an efficiency improvement from 3.0% (without CNTs) to 4.1% [182]. Further, unlike other common electron transport materials, CNTs do not degrade upon exposure to water or oxygen. Further, Lu and co-workers [183] added N-doped MWCNTs into active layers based on polythieno[3,4-b]-thiophene-co-benzodithiophene (PTB7) and PC71BM, leading to an efficiency increase from 7.3% to 8.6% by raising the MWCNT concentration. The improvement was ascribed to better light coupling, exciton dissociation and charge transport due to the presence of the nanotubes into the active layer.

Overall, the recent developments in the dispersion techniques for CNTs and the better understanding of their role in PVCs has led to a strong increase in efficiency, from 0.04% in 2002 for a PSC with SWCNTs dispersed in P3OT as active layer to 8.6% for N-doped MWCNTs in PTB7/PC71BM as active layer. Nonetheless, preliminary works on PSCs incorporating either GO or CNTs in the active layer indicated better performance for those comprising GO. For instance, the effect of a thiophene derivative, PTM21, covalently grafted to the surface of GO and MWCNTs on the cell efficiency was investigated [184]. Albeit the performance was improved upon addition of both carbon nanofillers, the highest efficiency was attained with 0.3 wt % GO, ascribed to the larger surface contact area, improved charge transport, and better dispersibility of the GO layers compared to the CNTs. Moreover, the amphiphilic character of GO facilitates the device processability in aqueous solutions, while CNTs present a hydrophobic nature and are insoluble in most of the common solvents.

## 6. Conclusions and Future Perspectives

Currently, photovoltaic technology is regarded as a part of the solution to the growing energy challenge and as a key component of future global energy production. In this work, a brief description of the state of art on photovoltaic cells has been provided. The different technologies developed up to date have been divided into four generations, and the characteristics, advantages and limitations of each generation along with the most recent investigations have been discussed in detail. A summary of these technologies, production methods, characteristics, and efficiencies attained is given in Table 1. 

Most 1GEN (m-Si, p-Si and GaAs) and 2GEN (a-Si, μc-Si, CdTe/CdS, and CIGS) technologies are highly standardized and have undergone few changes in recent years; they exhibit high efficiencies (20–25%) and are typically expensive, though there has been a reduction in the cost of silicon-based cells. On the other hand, the majority of 3GEN (QDs, perovskite, PSCs, DSSCs), as well as 4GEN (polymers combined with metal nanoparticles, CNTs, G or its derivatives) technologies are in states very close to the so-called “basic research”; laboratory prototypes that lead to good results have been developed though they have not been implemented at an industrial scale yet (efficiencies 10–15%). However, the 3GEN multi-junction cells are already commercial, and have achieved very high energy conversion rates (>40%), thus becoming the best alternative if efficiency is sought. 4GEN cells based on CNTs, G or its derivatives are in a state of early-research, hence constitute a very promising field for investigation. The versatile nature of such carbon nanostructures allowing to incorporate them throughout the PSC architecture, including transport layers, active layer, and electrodes, with the aim to attain inexpensive stable devices with improved performance. Both G and CNTs have been shown to be an effective, solution processable, replacement for traditional transport layers such as PEDOT:PSS. Furthermore, CNT doping has been proved to be effective for tuning the charge transport within the active layers. Additionally, there is also promise in the fabrication of hybrid architectures involving metal oxide/carbon nanostructures as transport layers in DSSCs. 

Despite the increasingly evident enhancements in PSC performance due to the addition of the carbon nanostructures, they have not been introduced into the market yet, since several issues have to be addressed. (1) New approaches that enable to synthesize high-purity and high-quality CNT or G thin films with controlled morphology and electronic properties need to be developed, since the purity, quality, band-gap, and morphology of the carbon nanostructures conditions the PSC performance. In the case of TCEs, solution processable films with an optimum balance between sheet resistance and transparency are required. Further, the functionalization and ultrasonication processes used during the fabrication of PSCs lead to a remarkable drop in the electrical conductivity of the carbon nanomaterials. Thus, the electrical properties of most conjugated polymer/carbon nanotube composites do not fulfil the requirements for TCEs and counter electrodes in PVCs. (2) The actual specific surface area of carbon-based nanomaterials is smaller than the predictions due to their strong agglomeration tendency by means of π-π stacking interactions, and the mixture with polymers makes the issue worse. Hence, novel synthetic methods to prevent aggregation are sought. (3) Novel doping or functionalization approaches compatible with the fabrication process of PSCs have to be designed to attain higher stability, improved charge transport and tunable energy levels in carbon-based nanomaterials. (4) New inexpensive techniques that enable the synthesis of CNTs and G at a large scale are need. Despite some improvements have been attained in this direction, the current methods are gravely limited by their low yields, and this needs to be addressed prior to their use in commercial applications.

It is envisaged that in the next future and after comprehensive research on the field, 4GEN PSCs incorporating carbon-based nanomaterials would offer high performance levels to rival those of traditional silicon-based cells, thus providing a new outlook for the solar energy industry.

## Figures and Tables

**Table 1:** Summary of PV technologies.

GEN	Technology	Production Method	Characteristics	Efficiency (%)	Reference
1GEN	m-Si	Czochralski	Expensive, stable	24.4	[8,15]
1GEN	p-Si	Siemens	Low cost, high defect content	19.9	[15,16]
1GEN	GaAs	Expitaxial growth	Expensive, good design control	18.4–28.8	[15,19,20]
2GEN	a-Si	Large-area deposition	Non-toxic, short life cycle	10.2–12.7	[15,24]
2GEN	μc-Si	Roll-to-roll	Low defect content, good degradability	11.9–14.0	[13,22]
2GEN	CIGS	Deposition, co-evaporation	Tuneable band gap	22.3	[13,26,27]
2GEN	CdTe	Deposition	High temperature tolerance, low fooling	21	[13]
3GEN	DSSC	Roll-to-roll,	Work in low-light conditions, robustness	5.0–20.0	[30]
3GEN	QDs	Solution casting	Efficient conductivity	11.0–17.0	[52]
3GEN	OPSCs	Solution casting	High work function, thermally stable	9.7–11.2	[13,77]
3GEN	PVSC (CH3NH3PbI3)	Sputtering/ printing	Cheap, simple	21.1–21.6	[13,87]
3GEN	MJ ^1^	Stacking	Wide range of design, Challenging manufacture	35.8	[13,105,111]
3GEN	IMM ^2^	Monolithic growth	Cheap, high band gap	40–44.4	[13,105,114,185]
4GEN	BHJ ^3^ PSC ^4^ with GO/PEDOT:PSS	Solution casting	Reproducible and stable	4.28	[123]
4GEN	PSC with G/PEDOT:PSS	Solution casting	Good functionality	2.82–11.8	[121,153]
4GEN	PVSC ^5^ with Li-GO	Spray deposition	Stable, long lifetime	1.07–11.14	[186]
4GEN	PVSC with rGO/PEDOT:PSS	Solution casting	Long lifetime, reduced elec.-hole recombination	5.7–11.95	[129,152,157,158]
4GEN	PSCs with B-doped CNTs	Solution casting	Improved electron transport	4.1–8.6	[182]

^1^ MJ: multi-junction; ^2^ IMM: inverted methamorphic multijunction; ^3^ BHJ: Bulk heterojunction; ^4^ PSC: polymer solar cell; ^5^ PVSC: perovskite.
